# Biologically Active Preparations from the Leaves of Wild Plant Species of the Genus *Rubus*

**DOI:** 10.3390/molecules27175486

**Published:** 2022-08-26

**Authors:** Łukasz Kucharski, Krystyna Cybulska, Edyta Kucharska, Anna Nowak, Robert Pełech, Adam Klimowicz

**Affiliations:** 1 Department of Cosmetic and Pharmaceutical Chemistry, Pomeranian Medical University in Szczecin, PL-70111 Szczecin, Poland; 2 Department of Microbiology and Environmental Chemistry, Faculty of Environmental Management and Agriculture, West Pomeranian University of Technology, PL-71434 Szczecin, Poland; 3 Faculty of Chemical Technology and Engineering, Department of Chemical Organic Technology and Polymeric Materials, West Pomeranian University of Technology, PL-70322 Szczecin, Poland

**Keywords:** leaves of *Rubus idaeus* L., leaves of *Rubus fruticosus* L., biologically active preparations, bioactive compounds, antioxidative potential, antimicrobial activity

## Abstract

The plants of the genus *Rubus* (*R.*) are applied as antiseptic agents in the treatment of skin diseases. Despite the great interest in plants of this genus, there are few reports on the antioxidant and biological activities of preparations obtained from the leaves of these plants. Therefore, we decided to evaluate the antioxidant activity of preparations from leaves of wild plant species of the genus *Rubus* using the frequently applied DPPH, ABTS, and FRAP methods, as well as to determine the total polyphenol content using the Folin–Ciocalteau method and perform qualitative evaluation by gas chromatography–mass spectrometry (GC-MS). The bactericidal and fungicidal activities of the obtained preparations were evaluated by applying laboratory tests: using the disc and the well methods based on the standards EN 13697:2019, EN 13697:2015, and EN 1500:2013. Microbiological tests of the plant preparations against bacteria, fungi, and yeasts isolated from the environment and against reference strains were performed. Moreover, antimicrobial testing of antibiotics against the tested strains was performed for comparison. The n-octanol/water partition coefficient of the obtained preparations was determined by the shake-flask method to determine their lipophilicity. According to the results, a high content of polyphenols and other antioxidant and biologically active compounds can be thought of as the parameter responsible for the effective activity of plant preparations obtained from wild plant species of the genus *Rubus*. The methods for determining bactericidal and fungicidal activity clearly demonstrates that preparations with reduced ethanol content exhibit bactericidal and fungicidal activity on surfaces. Testing of hand disinfection by means of rubbing with the preparations confirmed their antimicrobial activity against *Escherichia coli* K12 NCTC 10538. The obtained results show that the tested preparations exhibit on average two times lower activity against the reference bacterial strains than comparable antibiotics. The preparations obtained from the leaves of *R. idaeus* L. and *R. fruticosus* L. could complement classical antibiotics. While environmental bacteria showed a similar response to the preparations and antibiotics, their sensitivity was about one-third less than that of the reference strains. Our studies have shown that the obtained preparations are highly hydrophilic (logP < 0). Thus, these preparations can only be used in lipid bilayers in the aqueous core of liposomes, not in the lipid envelope.

## 1. Introduction

The skin is a physical, chemical and biological barrier against external factors [1,2,3,4]. Structurally, the skin provides a favorable habitat for the growth of microorganisms, including those involved in the development of many diseases [4,5,6,7,8,9]. In recent years, there has been growing interest in plant-based preparations derived from wild plants. Preparations topically applied to the skin (apart from their antioxidant activity, which is extremely important in the prevention of cell aging processes) are characterized by antimicrobial activity against pathogenic microorganisms occurring on the skin [5,6,10,11,12,13,14,15,16].

Reactive oxygen species (ROS), a major factor in skin aging, cause disruption of cellular metabolism by interfering with the structure of proteins, lipids, and DNA [6]. The consequence of ROS activity is increased oxidative stress, which influences the process of cell degradation [16,17,18,19]. Reactive oxygen species are thought to be responsible for many cell disorders through their chemical attacks on biostructures. ROS exist both endogenously and exogenously for living organisms. These structures are constantly made through human metabolism due to oxidation [11]. It has been reported that 1–3% of the oxygen molecules reduced in mitochondria may be the reason of superoxide radical formation [12]. Antioxidant compounds block oxidation chain reactions by scavenging free radicals in the metabolism. In the past few decades, investigation about new natural sources of antioxidants has become more popular.

Many plant species are of economic and medicinal importance due to their rich content of essential oils. These plants show antispasmodic, anti-inflammatory, antioxidant, antidiarrheal, antiviral, antibacterial, and antifungal activities. Although the phenolic compound content, antioxidant and antimicrobial activities of many plants has been well investigated, little information is available on preparations derived from wild plant species. It is clear that most green plants, vegetables, and fruits are major sources of natural antioxidants. Consuming these antioxidants in the human diet is a reasonable way to reduce brain dysfunction, cardio-vascular disease, cataracts, and risk of cancers [11,13,14,20,21,22,23,24,25,26,27,28,29,30,31,32].

*R. idaeus* L. and *R. fruticosus* L. are perennial plants that belong to the genus *Rubus *spp. of the family *Rosaceae*. The fruits of these plants are often used as an important source of valuable minerals. In traditional medicine, the leaves of these plants are applied in infusions because of their diuretic, diastolic, antidiabetic, and anti-diarrheal effects. Compresses or lotions made from the leaves of these plants are used to treat various skin diseases thanks to their strong astringent effect [17,18,19,20,21,22,23,24,32,33,34,35,36]. Methanolic and ethanolic as well as aqueous–ethanolic extracts from the fruits, shoots, and all parts of different *Rubus idaeus* varieties are effective against *Streptococcus typhi*, *Streptococcus pneumoniae*, *Staphylococcus aureus*, *Streptococcus epidermidis*, *Moraxella catarrhalis*, *Haemophilus influenzae*, *Helicobacter pylori*, *Klebsiella pneumoniae*, *Enterococcus faecalis*, *Bacillus subtilis*, and *Escherichia coli* [17]. The seed oil of the plants of the genus *Rubus* contains vitamins, steroids, and lipids, while, minerals, flavonoids, glycosides, terpenes, acids, and tannins have been identified in the aerial parts. Therefore, the seed oils and extracts obtained from aerial parts of these plants exhibit a variety of pharmacological activities, such as antioxidant, anticancer, anti-inflammatory, antibacterial, antidiabetic, antidiarrheal, and antiviral. As these plants are famous for their aerial parts and seeds of medicinal, cosmetic, and nutritional value, and are concentrated sources of valuable nutrients and bioactive components of therapeutic importance, we decided to study the antioxidant and antimicrobial activity of preparations from the leaves of these plants [17,20,21,22,23,24,37,38,39,40].

In the present study, we investigated the antioxidant potential of two preparations, preparation 1 (P1) obtained from the leaves of *R. idaeus* L. and preparation 2 (P2) obtained from the leaves of *R. fruticosus* L. The biocidal and bactericidal activity of P1 and P2 was evaluated against reference bacterial strains, fungi, and yeasts. In addition, we tested hand disinfection by means of rubbing with the different preparations. To determine the lipophilicity of the preparations, the n-octanol/water partition coefficient was determined by the shake-flask method. Moreover, the overall purpose of this study was to obtain plant preparations with reduced ethanol content, because ethyl alcohol used at a concentration of 70 g/100 mL is currently registered as a raw material for the production of various preparations. Ethanol is used in the preparation of medicines as a pharmaceutical raw material, and must be used in accordance with the Pharmaceutical Law for admission to trading by the Registration Office. According to the mentioned methods, the preparations from leaves of *R*. *idaeus* L. and *R. fruticosus* L. showed good potential antioxidant and radical-reducing activity. The quantitative methods for determining the bactericidal and fungicidal activity of chemical disinfectants and antiseptics clearly demonstrated that the resulting preparations exhibit bactericidal and fungicidal activity on surfaces. Testing of hand disinfection by means of rubbing with the preparations confirmed their antimicrobial activity against *Escherichia coli* K12 NCTC 10538. The effect of preparations applied on the skin depends on the activity of biologically active substances as well as on the ability of the active substances to penetrate the skin, especially through the so-called intercellular cement. The intercellular cement is hydrophobic and lipophilic, consisting of dead *Stratum corneum*. Liposomes are spherical vesicles consisting of one or more lipid bilayers arranged concentrically. The core of liposomes is a droplet of water. Lipophilic vehicles can increase the diffusion of substances by mixing with intercellular lipids. Lipophilicity studies showed that both the P1 and P2 obtained preparations are highly hydrophilic (logP = −0.18 in the case of P1 and −0.19 in the case of P2). Thus, these preparations can be found in lipid bilayers in the aqueous core of liposomes, not in the lipid envelope.

## 2. Results

### 2.1. Phytochemical Components of Preparations Obtained from the Leaves of Rubus idaeus L. and Rubus fruticosus L.

The plant preparations from the leaves of *Rubus idaeus* L. and *Rubus fruticosus* L. were analyzed using gas chromatography–mass spectrometry (GC-MS).

In Table 1, the components of the preparations and their potential biological activities are shown.

The major components (>1% of the total peak area) of the plant preparation from leaves of *Rubus idaeus* L. were 4H-pyran-4-one, 5-(hydroxymethyl)furfural, pyrogallol, hexadecanoic acid, and linoleic acid methyl ester. The same compounds were identified in the preparation from the leaves of blackberry (*Rubus fruticosus* L.), along with 2-hydroxy-5-methylbenzaldehyde and quinic acid. The other constituents (<1% of the total peak area) of preparations P1 and P2 were 2-hexenal, 2-heptanone, 2-hexanol-3-methyl, 4-heptanol-3-ethyl, 3-hexanol-5-methyl, 2,4-heptadienal, and 2-nonanone. Furthermore, analysis revealed the presence of two acids, n-decanoic acid (only in P2) and dodecanoic acid. For details, see supplementary data: GC-MS chromatogram of preparation 1 (P1) obtained from leaves of the *Rubus idaeus* L. (Figure S1); GC-MS chromatogram of preparation 2 (P2) obtained from leaves of the *Rubus fruticosus* L. (Figure S2), and the structures of the compounds identified in the tested preparations obtained from the leaves of *R. idaeus* L. and *R. fruticosus* L. (Table 1, Figure S3).

### 2.2. ATR-FTIR Studies

**Figure 1:**
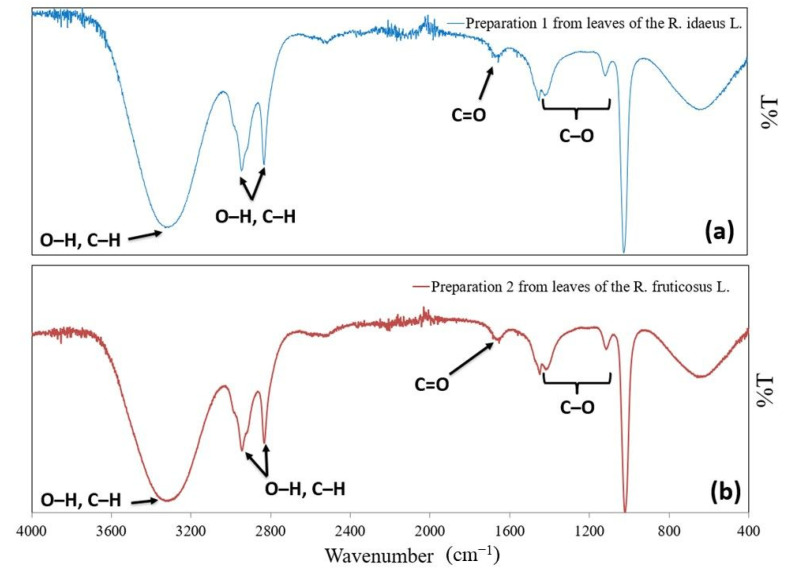
ATR-FTIR absorption spectra of (**a**) preparation 1 obtained from the leaves of *R. idaeus* L. and (**b**) preparation 2 obtained from the leaves of *R. fruticosus* L.

Figure 1 shows the IR spectrum of preparation 1 obtained from the leaves of *R. idaeus* L. (a) and preparation 2 obtained from the leaves of *R. fruticosus* L. (b).

In the IR spectra of the P1 and P2 there is an absorption band at a wavenumber of about 1655 cm^−1^, which is characteristic of the carbonyl group derived from ketones (2-heptanone, 4H-pyran-4-one, 2-nonanone) and ester (linoleic acid methyl ester). There are bands at wavenumbers of around 3300, 2940, and 2830 cm^−1^, attributed to the hydroxyl group’s stretching vibration (O-H). These groups can be derived from the following compounds: 2-hexanol-3-methyl, 4-heptanol-3-ethyl, 3-hexanol-5-methyl, 4H-pyran-4-one, 5-(hydroxymethyl) furfural, pyrogallol, dodecanoic acid, hexadecenoic acid, and 2-hydroxy-5-methylbenzaldehyde, with quinic acid and n-decanoic acid present only in preparation 2. The occurrence of the absorption bands at the respective wavenumbers (i.e., around 3300, 2940, and 2830 cm^−1^) is attributed to the stretching vibrations originating from the C-H carbon atoms. The IR spectrum shows absorption bands in the range from 1430 to 1110 cm^−1^, derived from the single-molecule stretching bonds of 4H-pyran-4-one and 5-(hydroxymethyl)furfural (Figure 1) [7,49,50,51,52,53].

### 2.3. Evaluation of Free Radical Scavenging Activity

Table 2 presents the antioxidant activity and total polyphenol content of the preparations obtained from *Rubus idaeus* L. and *Rubus fruticosus* L. leaves, with measurement carried out using the DPPH, ABTS, FRAP, and Folin–Ciocalteau methods.

Ethanolic and ethanol–water extracts obtained from plant materials are characterized by their antioxidant potential [52,54,55,56,57]. The ethanol extracts from leaves, buds, and bark of *Cinnamomum cassia* exhibit remarkable antioxidant potential, with a total polyphenol content ranged from 6.313 to 9.534 g GA/100 g dry weight of materials (DW). The highest polyphenol content is found in the ethanol extracts of bark (9.534 g GA/100 g DW), followed by the leaves (8.854 g GA/100 g DW) and buds (6.313 g GA/100 g DW) peroxides [46,56]. These results reveal that in the 95% ethanol extracts of leaves, the total polyphenol content (8.854 g/100 g DW) was about two times the total flavonoid content (3.348 g/100 g DW). The 50% ethanol extracts of leaves from Chinese *Cinnamomum cassia* possess a total phenolic content of 1558.7 μg GA/g DW and total flavonoid content of 981.1 μg/g DW. Polyphenols are secondary metabolites existing throughout plants; they have important attributes, including scavenging free radicals and decomposing peroxides [46,56].

In vitro antioxidant activity of 95 and 50% ethanol extracts of the different samples of *Tephrosia purpurea* collected in the summer (April), rainy (August), and winter (December) seasons were determined by Edewor et al. [58]. *Tephrosia purpurea* showed a wide variation in IC_50_, ranging from 65 to 156 µg/mL [58].

Extracts of *Rosehips* are rich in compounds having antioxidant properties, such as vitamin C, carotenoids, and phenolics. Extract of *Rosehips spinosissima* in 95% ethanol shows higher phenolic content and antioxidant activity compared to extract in 50% ethanol. Moreover, the total antioxidant capacity of water and ethanolic extract of *Rosehips* was evaluated by FRAP assay. The obtained range of FRAP values proved similar to those found by other authors, including Souza et al. [30], Oszmiański et al. [50], Mostafa et al. [51] and Rojas-Vera et al. [52].

The results presented in Table 2 show that higher antioxidant activity (for all methods) and higher total polyphenol content were observed for plant preparations obtained from *Rubus fruticosus* L. leaves: 3.43 ± 0.02 mg trolox/g raw material for the DPPH method, 20.01 ± 0.20 mg trolox/g raw material for the ABTS method, 7.39 ± 0.13 mg trolox/g raw material for the FRAP method, and 7.55 ± 0.43 mg GA/g raw material for the Folin–Ciocalteau method. The antioxidant activity and total polyphenol content of the preparation obtained from *Rubus idaeus* L. leaves was characterized by the DPPH, ABTS, FRAP, and Folin–Ciocalteau methods and were 3.38 ± 0.03 mg trolox/g raw material, 17.89 ± 1.07 mg trolox/g raw material, 6.13 ± 0.17 mg trolox/g raw material, and 6.52 ± 0.57 mg GA/g raw material, respectively; see Table 2.

### 2.4. Surface Disinfection Tests of the Plant Preparations by Standard EN 13697:2019

Table 3 and Table S2 show the results of surface disinfection tests of preparation 1 (P1) obtained from the leaves of *R. idaeus* L. and preparation 2 (P2) obtained from the leaves of *R. fruticosus* L. according to the EN 13697:2019 standard.

Preparation 1 (P1) obtained from the leaves of *R. idaeus* L. and preparation 2 (P2) obtained from the leaves of *R. fruticosus* L. were assessed using the surface disinfection test. For this test, the ‘pass’ criteria was a 99.9% reduction (LR > 3) in test organism viability. The results for the surface disinfection tests (Table 3) showed that P1 and P2 (at a concentration of 60 g/100 mL) showed biocidal activity against all tested strains both after contact time (60 ± 10 s) and after 300 ± 10 s. The percentage of microbial reduction (% reduction) was above 99.9, and the log reduction was above 3; see Table 3.

### 2.5. Surface Disinfection Tests of Plant Preparations by Standard EN 13697:2015

Table 4 and Table S3 shows the results of surface disinfection tests of preparation 1 (P1) obtained from the leaves of *R. idaeus* L. and preparation 2 (P2) obtained from the leaves of *R. fruticosus* L. against *Staphylococcus aureus* ATCC 6538 and *Pseudomonas aeruginosa* ATCC 15442.

Preparation 1 at a concentration of 35–70 g/100 mL was shown to be especially active on surfaces against *Staphylococcus aureus* ATCC 6538, and at a concentration of 42–70 g/100 mL against *Pseudomonas aeruginosa* ATCC 15442. Preparation 2 at a concentration of 35–70 g/100 mL was shown to be especially active on surfaces against *Staphylococcus aureus* ATCC 6538 and *Pseudomonas aeruginosa* ATCC 15442—Table 4.

### 2.6. Testing of Hand Disinfection by Means of Rubbing in Plant Preparations According to EN 1500:2013

Table 5 shows the statistical analysis of the preparations obtained from the leaves (P1 and P2) and the reference preparation (PR) according to EN 1500:2013 when using 6 mL of the corresponding preparation against the test strain of *Escherichia coli* K12 NCTC 10538; Supplemental Table S1 presents the sorted values for the statistical test.

The results of the hand disinfection test showed that the critical value for the Wilcoxon test (in the case of PRP1 and PRP2) was 52, which is smaller than the highest mean value of 53 (in the case of PRP1 and PRP2) and does not increase above the *LR* value of 0.60. The value of 53, the highest mean value in the case of PRP1, is −0.22, and 0.09 in the case of PRP2, meaning that the hypothesis of lower activity of test preparations 1 and 2 relative to the RP can be rejected. Thus, we conclude that the tested preparations do not have lower activity than propan-2-ol (Table 5 and Table S1). Testing of hand disinfection by means of rubbing with the preparations in a volume of 6 mL confirmed their antimicrobial activity against *Escherichia coli* K12 NCTC 10538 after a contact time of 60 s according to EN 1500:2013. These results show that the tested preparations do not have lower activity than the reference preparation (see Table 5 and Table S1).

### 2.7. Biological Activity of Dry Preparations and Antibiotics

**Figure 2:**
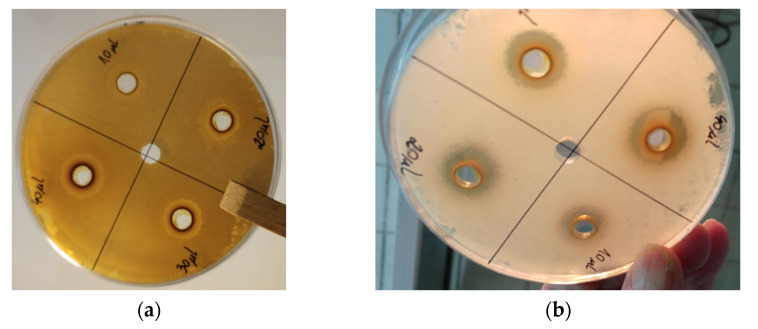
Methods of application of plant preparation to Trypticase Soy Agar using the well method against (**a**) *Escherichia coli* and (**b**) *Bacillus sp.*

Our evaluation of the methods of plant preparations applied to medium showed that the well method should be used. This is probably due to the different diffusion parameters of the active substances contained in P1 and P2 and their sorption in the blotting paper (Figure 2). The tests described in Section 4.11.1 show that using preparations 1 and 2 at the amount of 40 µL results the highest growth inhibition zone of microorganisms. Therefore, the preparations were used at 40 µL for microbiological evaluation.

For antibiotics, the disc method is suitable and commonly used. Therefore, microbiological evaluation of antibiotics against the tested strains by the well method was not conducted.

**Figure 3:**
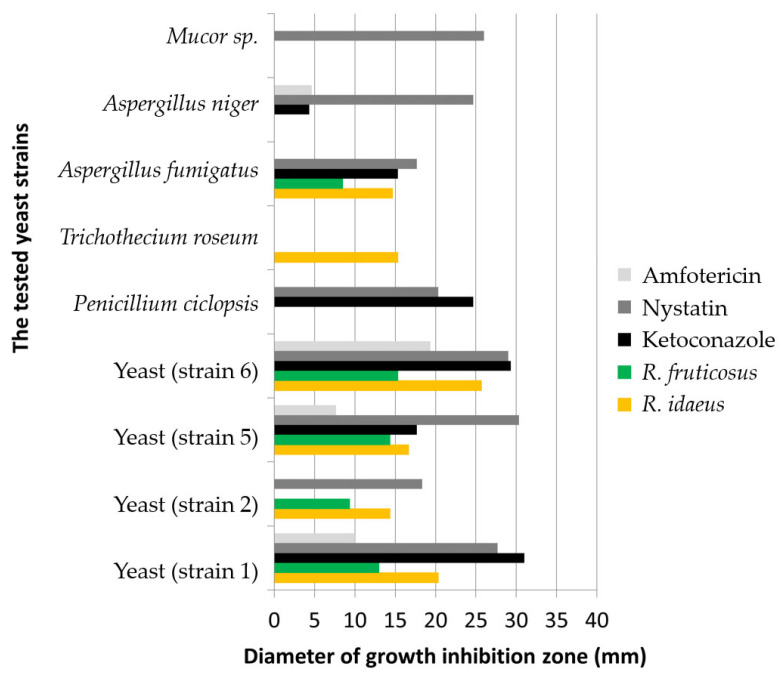
Interaction of P1, P2, and antibiotics with isolated yeast and fungal strains.

**Figure 4:**
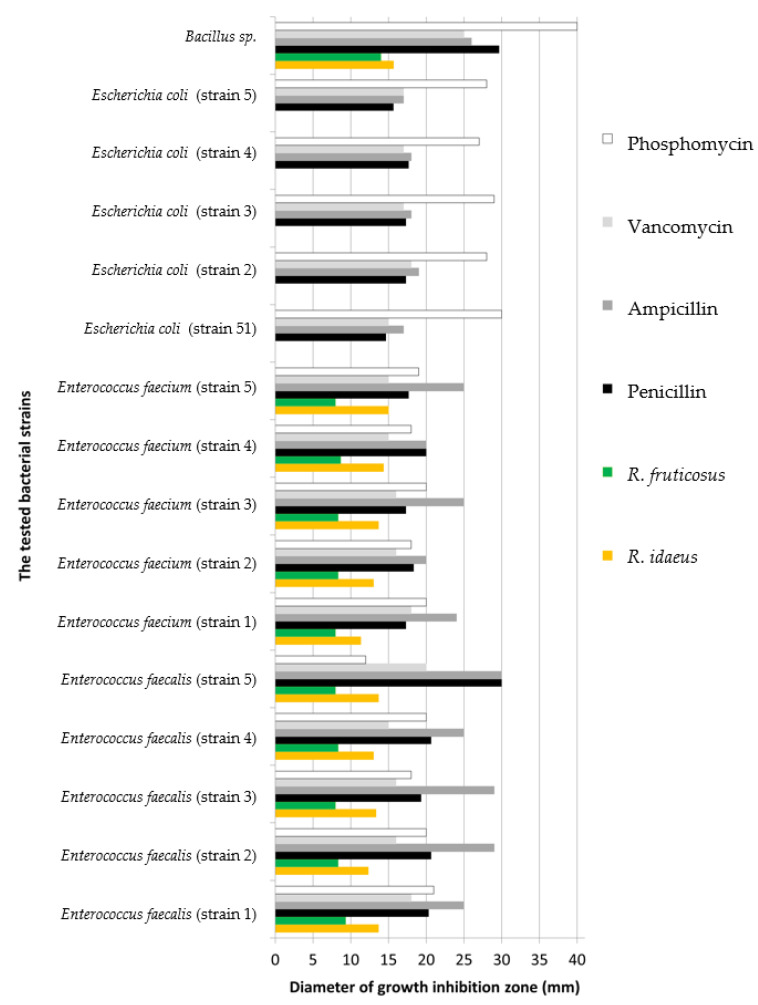
Interaction of P1, P2, and antibiotics with the isolated bacterial strains.

Figure 3 and Figure 4 show the effects of the plant preparations obtained from *Rubus idaeus* L. and *Rubus fruticosus* L. leaves on microorganisms isolated from food industry environments and their surroundings.

The plant preparation of *R*. *idaeus* leaves was active against seventeen microorganisms and *R. fruticosus* against sixteen tested strains of microorganisms. No activity was observed against *Penicillium ciclopsis*, *Aspergillus niger*, *Mucor* sp., or *Escherichia coli*, and in the case of blackberry preparation, additionally against *Trichothecium roseum* strain.

The effect of antibiotics against isolated bacterial strains was stronger than that of the preparations. All types of antibiotics were active against the isolated bacteria (Figure 4). However, there was no activity of the antibiotics Amphotericin against all fungi and one strain of yeast, Nystatin against one fungal strain, and Ketoconazole against two fungi and one strain of yeast. The plant preparations were active against all yeasts, and against *Aspergillus fumigatus*. Of all tested preparations and antibiotics, only P1 was active against *Trichothecium roseum* (Figure 3). Preparations 1 and 2 were tested for antimicrobial activity against *Escherichia coli* strains, with no growth inhibition zones observed against these microorganisms.

Table 6 shows the zones of growth inhibition induced by plant preparations and antibiotics against thirteen model bacterial strains.

Similar antibacterial activity of both tested plant preparations was found against thirteen reference bacterial strains (Table 6). The diameter of zones of growth inhibition against the tested bacterial strains ranged from 6.3 to 21.7 mm for the preparation obtained from raspberry leaves and from 6.0 to 19.7 mm for the preparation obtained from blackberry. The highest antibacterial activity (growth inhibition zone 21.7 mm in the case of P1 and growth inhibition zone 19.7 mm in the case of P2) was demonstrated against the *Streptococcus intermedius* reference strain.

The raspberry preparation had slightly higher activity against ten bacterial strains than the blackberry preparation, which showed slightly higher zones of inhibition against *Enterococcus faecalis* (18.3 mm) and *Staphylococcus aureus* (A) (18.0 mm). Against the *Enterococcus faecalis* strain, both plant preparations and the Ampicillin antibiotic showed similar values of inhibition zones, with no statistically significant differences (15.0–18.3 mm). Against the *Bacillis thuringiensis* strain, P1, P2, and the Doxycycline antibiotic were characterized by similar values of growth inhibition zones and no statistically significant differences were shown (12.3–15.0 mm). However, against the *Klebsiella pneumoniae* strain, the raspberry leaf preparation had higher activity than Doxycycline and showed a statistically significant larger zone of growth inhibition (8.3 mm) than the tested antibiotic (5.7 mm).

### 2.8. Measurement of Lipophilicity

**Figure 5:**
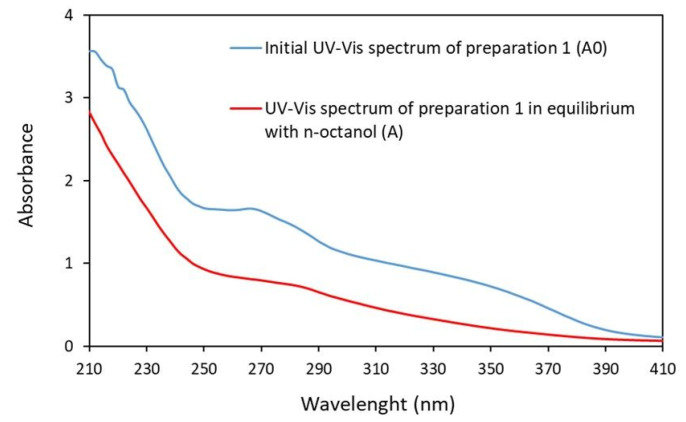
An example of the UV-Vis spectrum of the preparation obtained from the leaves of *Rubus idaeus* L.

Our results show that the values of the partition coefficient determined by the shake-flask method for the preparations obtained from the *Rubus idaeus* L. (P1) and *Rubus fruticosus* L. leaves (P2) were −0.18 ± 0.001 and −0.19 ± 0.002, respectively; see Figure 5. Lipophilicity studies show that both preparations are highly hydrophilic.

## 3. Discussion

The extracts of plant materials might be a potential antioxidant supplement for food and pharmaceutical products, and could be used to protect the foods against oxidative deterioration. The results of this study indicate that preparations made from *Rubus genus* leaves have effective ABTS radical scavenging activities, similar to standard antioxidants, i.e., butylated hydroxyanisole (BHA), butylated hydroxytoluene (BHT), and α-tocopherol. In addition, studies conducted by other authors have shown that extract obtained from leaves of *R. fruticosus* can be a potent antioxidant for stabilization of sunflower oil. Their results showed the highest efficiency of 1000 ppm of this extract, followed by BHT and BHA. Methanolic and ethyl acetate extracts were prepared from young leaves of *Pistacia atlantica Desf*., which were collected during the spring, and their antioxidant, chelating, and scavenging activities were evaluated. Extracts obtained from leaves of *Pistacia atlantica Desf*. showed strong antioxidant activity compared to synthetic antioxidants such as BHA, BHT, and α-tocopherol; see Table 2 [7,59,60].

The high polyphenol content and antioxidant capacity of raspberry, blackberry, and Andes berry (*Rubus glaucus* Benth) suggest that these fruits could be a rich source of natural pigments, nutraceuticals, and natural antioxidants. The total polyphenol content (evaluated by the Folin–Ciocalteau method), the antioxidant activity (measured by ABTS radical scavenging capacity), and ferric reducing antioxidant power (FRAP) have previously been determined in extracts of Andes berry fruit. The total polyphenol content was 294 ± 37.2 mg GA/100g raw material, while the antioxidant activity and ferric reducing antioxidant power was 2.01 ± 0.12 and 4.50 ± 1.22 mmol trolox/100 g raw material, respectively [42]. The DPPH radical scavenging activity and the total polyphenol content of fruit extracts from *Rubus* genera was tested by Benvenuti et al. [44]. Total polyphenols ranged from 140.6 to 888.5 mg/100 g fresh weight. The average EC50 values for *Rubus fruticosus* and *Rubus idaeus* were 6.4 and 8.2 mg fresh weight, respectively. Their results indicate that the tested fruits are good sources of natural antioxidants [44]. In view of the pharmacological interest in phenolic substances, Lucas et al. [43] determined the total polyphenols in the berries of several cultivars of *Rubus*. Moreover, the in vitro antiradical activity of crude extracts on chemically generated superoxide radicals as well as their inhibitory activity towards the xanthine oxidase enzyme were studied. All the crude extracts examined showed activity towards chemically-generated superoxide radicals, a certain inhibitory activity towards xanthine oxidase, and were characterized by their polyphenol content [43]. The polyphenol, vitamin C, and anthocyanin contents of the berry fruit species belonging to the genera *Rubus* have been evaluated as well. These compounds have interesting properties, such as anti-inflammatory, aromatic, healing, antioxidative, and capillary vessel protecting qualities. This justifies a broad use of berry fruits for both food purposes and as a source for the pharmaceutical and cosmetic industries [43,45]. The total polyphenol content in the obtained preparations evaluated in our study was high (Table 2). This level is higher than previously reported by Garźon et al. [42], Costantino et al. [43], Rotundo et al. [45], and Benvenuti et al. [44]; see Table 2.

The common biological activities of the abundant compounds of preparations from *Rubus idaeus* L. and *Rubus fruticosus* L. leaves are antioxidant, anti-inflammatory, antibacterial, and antifungal [7,42,43,44,45,46,47,48,54,59,61]; see Table 1. The obtained results in our study indicate the possibility of using preparations from the leaves of *Rubus idaeus* L. and *Rubus fruticosus* L. as cosmetics applied topically to the skin. The preparations obtained from leaves, thanks to their high content of polyphenols and other biologically active compounds (Table 1 and Table 2), inhibit the formation of reactive oxygen species (ROS) [7,9,25,47,61], and are therefore appear promising as antioxidant delivery systems.

The pulverized seeds of *Azanza garckeana* were used by Momodu [48] to obtain methanol and aqueous extracts, which were then subjected to GC-MS analysis. The presence of compounds with antioxidant activity were methyl oleate, palmitic, myristic, and hexadecanoic acids, and 11-octadecenoic acid methyl ester. The extract from seeds of *Azanza garckeana* has useful constituents that can be exploited for health benefits [48]. Similarly, in our previous study [6,7,61] linoleic acid, methyl ester, and hexadecanoic acid (palmitic acid) were identified as major components of leaf extract. The presence of methyl esters of fatty acids (methyl palmitate and methyl oleate) has been confirmed by others in the extracts of dried and fresh leaves of plants [46] and essential oils from *E. angustifolium* [33] and *E. hirsutum* [61,62,63]. Among the major components identified by Canli et al. [49] using GC-MS with ethanol extracts of plants, fatty acids were a large group [49]. Extracts from *R. perrobustus*, *R. wimmerianus*, *R. pedemontanus*, and *R. grabowskii* leaves were assessed regarding their phenolic compound profiles and antioxidant activity. Thirty-three phenolic compounds were detected (hydroxycinnamic acids, flavonols, ellagic acid derivatives, and flavones). Ellagic acid derivatives were the predominant compounds in the analyzed leaves, especially sanguiin H-6, ellagitannins, lambertianin C, ellagitannins, and casuarinin. The content of polyphenolics was significantly correlated with the antioxidant activity of the analyzed extracts. There is clear potential for the utilization of blackberry leaves as a food additive, medicinal source, or herbal tea [50]. Ethanolic extracts from leaves had potent activity against both Gram-positive and Gram-negative bacteria [41]. Unsaturated aldehyde (2,4-heptadienal) is known to exert antimicrobial and antifungal activity [43,60]. Furthermore, our analysis revealed the presence of two acids, n-decanoic acid (only in P2) and dodecanoic acid, both of which have previously been studied for their antifungal and antibacterial activity [43,60].

Preparation 1 at the concentration of 35–70 g/100 mL was shown to be especially active on surfaces against *Staphylococcus aureus* ATCC 6538, while a concentration of 42–70 g/100 mL was effective against *Pseudomonas aeruginosa* ATCC 15442. Preparation 2 at a concentration of 35–70 g/100 mL was shown to be especially active on surfaces against both *Staphylococcus aureus* ATCC 6538 and *Pseudomonas aeruginosa* ATCC 15442. For P1 at a concentration of 14–28 g/100 mL against *Staphylococcus aureus* ATCC 6538 and at a concentration of 14–35 g/100 mL against *Pseudomonas aeruginosa* AT CC 15442, the percentage of microbial reduction (% reduction) was high and remained above 98.1. For preparation 2 obtained from the leaves of *R. fruticosus* L. against *Staphylococcus aureus* ATCC 6538 and *Pseudomonas aeruginosa* ATCC 15442, the percent reduction was above 98.3; see Table 4. Antimicrobial activity has been confirmed in previous studies evaluating the antimicrobial activity extracts of *Rubus idaeus* and *Rubus occidentalis* shoots and leaves against Gram-negative and Gram-positive bacteria [17]. Ethanol–water extracts from varieties of *Rubus idaeus* “Ljulin”, “Veten” and “Poranna Rosa”, and *Rubus occidentalis* “Litacz” were evaluated to determine the range of their antimicrobial properties. An antimicrobial assay was performed using fifteen strains of bacteria. The antimicrobial activity of the extracts varied depending on the analysed strain of bacteria and the cultivar variety, with the exception of *Helicobacter pylori*, towards which the extracts displayed the same growth inhibiting activity. Two human pathogens, *Corynebacterium diphtheriae* and *Moraxella catarrhalis*, proved to be the most sensitive to raspberry extracts. The highest sensitivity of *Corynebacterium diphtheriae* to extracts from both *R. idaeus* and *R. occidentalis* may be due to its sensitivity to ellagic acid and sanguiin H-6 [17]. Moreover, antimicrobial activity by the fruit extracts of wild-type plants *R. moluccanus* and *R. alpesties* and *E. fraxinifolius* was shown against *B. subtitis*, *S. aureus* and *E. coli* as well [51].

## 4. Materials and Methods

### 4.1. Materials

Plant material (the leaves of *R. idaeus* L. and *R. fruticosus* L.) in the natural state was collected between May and October 2018 from the village of Siecino in Poland (West Pomeranian Voivodeship) by Dr. Łukasz Kucharski. The voucher specimens were deposited at the Department of Cosmetic and Pharmaceutical Chemistry, Pomeranian Medical University in Szczecin. The material was identified by Dr. Eng. of Agricultural Sciences Anna Nowak. The collected material was subjected to natural drying to a constant weight and then ground in an 8 mm mesh machine before extraction. 

### 4.2. Chemicals

The chemicals 2,2-diphenyl-1-picrylhydrazyl (DPPH), 6-hydroxy-2,5,7,8-tetramethylchroman-2-carboxylic acid (trolox), 2,2-azino-bis(3-ethylbenzothiazoline-6-sulfonic acid (ABTS), 2,4,6-tripyridyl-s-triazine (TPTZ) were purchased from Sigma Aldrich (Poland). Folin–Ciocalteu reagent was supplied by Merck (Darmstadt, Germany). Anhydrous sodium acetate, potassium persulfate, potassium acetate, ferric chloride, 36% hydrochloric acid, and ethyl alcohol were obtained from Chempur (Piekary Śląskie, Poland). All reagents were of analytical grade.

### 4.3. Plant Preparations from the Leaves of R. idaeus L. and R. fruticosus L.

The plant preparations from the leaves of *R. idaeus* L. and *R. fruticosus* L. were obtained as follows: 5.0 g of dried plant material and 45 mL of ethanol at a concentration of 70 g/100 mL were introduced into a conical flask and extraction was carried out using an ultrasound-assisted method with the use of an ultrasound bath at a frequency of 40 kHz for 1 h. The extracts were subjected to filtration on a pressure funnel through a Whatman paper filter (codified EEA03), thus obtaining the plant preparations preparation 1 (P1), obtained from the leaves of *R. idaeus* L., and preparation 2 (P2), obtained from the leaves of *R. fruticosus* L.

Ethanol was used as a polar solvent for this study because previous studies have shown that active compounds present in the leaves of *Rubus* are of a polar nature and that extracts produced with less polar solvents are largely devoid of activity [52].

### 4.4. Chemical Composition of Preparations from the Leaves of R. idaeus L. and R. fruticosus L.

The phytochemical components of the plant preparations from the leaves of *R. idaeus* L. and *R. fruticosus* L. were analyzed using GC-MS with a TRACE GC series apparatus equipped with a VOYAGER mass detector and using a DB5 capillary column (30 m × 0.25 μm × 0.5 μm). The following separation parameters were used for the analysis: a helium flow of 1.0 mL/min, detector voltage of 350 V, and sample chamber temperature of 240 °C. The thermostat temperature was increased according to the following program: isothermal at 50 °C for 1 min, increased at 8 °C/min, isothermal at 260 °C for 5 min, then cooled to 50 °C. The volume of the dispensed sample was 0.1 μL [53,54]. 

### 4.5. ATR-FTIR Studies

Analyses of preparations 1 and 2 from the leaves of *R. idaeus* L. and *R. fruticosus* L. was performed using total reflection-Fourier transform infrared spectroscopy (ATR-FTIR). An ATR unit obtained the spectra using a Thermo Scientific Nicolet 380 spectrometer (Thermo Fisher Scientific, Waltham, MA, USA). The recorded spectrum represented an average of 32 scans obtained with 4 cm^−1^. The spectra were collected in the wavenumber range of 4000–400 cm^−1^. The internal reflectance element (IRE) used in this study was an ATR diamond plate. The preparations were applied on the IRE.

### 4.6. Measurement of Antioxidant Capacity Using DPPH, ABT,S and FRAP Methods

Studies on the antioxidant activity of the plant preparations from the leaves of *R. idaeus* L. (P1) and *R. fruticosus* L. (P2) were carried out by free radical reduction (DPPH) [62] and (ABTS) [55] methods. Antioxidant activity was determined using the FRAP [56] method to determine the ferric reducing power assay. 

The analyses were performed on a Merck Spectroquant Pharo 300 apparatus at the following wavelengths λ: 517 nm in the case of the DPPH method, 734 nm in the case of the ABTS method, and 593 nm in the case of the FRAP method. For these methods, trolox was used as the reference substance and the antioxidant activity results obtained were expressed in mg trolox/g raw material. Measurement of antioxidative activity using the DPPH and ABTS methods was performed according to the procedure described previously [53]. Briefly, the antioxidant activity using DPPH was measured as follows: 2850 μL of an ethanol solution of DPPH radical was introduced into the tube (its absorbance at λ 517 nm was about 1.000 ± 0.020) along with 150 μL of the obtained plant preparation. The tube was incubated for 10 min at room temperature and spectrophotometric measurements were carried out at 517 nm in triplicate. The antioxidant activity using ABTS was measured as follows: 2500 μL of 7 mM solution of ABTS [53] and 25 μL of the obtained plant preparation were introduced into the spectrophotometric cuvette. The cuvette was incubated for 6 min at room temperature and the spectrophotometric measurement was carried out at 734 nm in triplicate.

Antioxidant activity using FRAP reagent was measured as follows: 2900 μL of prepared reagent and 100 μL of the obtained plant preparation were mixed in a cuvette. The cuvette was incubated for 8 min at room temperature and spectrophotometric measurements were carried out at 593 nm in triplicate [56]. To prepare the reagent, 25 mL of acetate buffer (0.3 M, pH = 3.6) was mixed with 2.5 mL of 2,4,6-tripyridyl-s-triazine solution (0.01 M TPTZ) in HCl (0.04 M HCl) and with 2.5 mL of FeCl_3_ solution (0.02 M). 

### 4.7. Measurement of Total Polyphenol Content Using Folin–Ciocalteau Method

Total polyphenol content of the plant preparations from the leaves of *R. idaeus* L. (P1) and *R. fruticosus* L. (P2) was determined with the Folin–Ciocalteau method as described previously [7,60]. The spectrophotometric measurements were performed on a Merck Spectroquant Pharo 300 apparatus at a wavelength of λ = 765 nm. Gallic acid (GA) was used as a reference substance, and the total polyphenol content was expressed in mg GA/g raw material. 

The total polyphenol content using Folin–Ciocalteau reagent was measured as follows: 1350 μL of distilled water and 1350 μL of sodium carbonate solution (0.01 mol/dm^3^) were introduced into a spectrophotometric cuvette with 150 μL of the prepared Folin–Ciocalteau solution [53] and 150 μL of the obtained plant preparation. The samples were incubated for 15 min at room temperature and spectrophotometric measurements were carried out at 765 nm and in triplicate. 

### 4.8. Surface Disinfection Tests of the Plant Preparations by Standard EN 13697:2019

The surface disinfection tests of preparation 1 (P1) obtained from the leaves of *R. idaeus* L. and preparation 2 (P2) obtained from the leaves of *R. fruticosus* L. by standard EN 13697:2019 included dilution of these preparations and their neutralization, in which microorganisms were treated with the preparations at concentrations 0.6 and 60 g/100 mL for time 60 ± 10 s and 300 ± 10 s, and at 20 ± 1 °C with the addition of a loading substance (bovine serum albumin). According to the guidelines of EN 13697:2019, in order to determine the bactericidal and fungicidal activity of the obtained preparations they should be diluted appropriately to test the antimicrobial activity of a given preparation from the active concentration (60 g/100 mL) to the inactive concentration (0.6 g/100 mL) [57].

First, the method was validated using the solution neutralization technique (water containing sodium thiosulfate, soy lecithin, and Polysorbate 80). The neutralizer used allowed the method to be validated (Table 7). The antimicrobial activity of the plant preparations was evaluated on discs against the following reference strains: *Staphylococcus aureus* ATCC 6538, *Pseudomonas aeruginosa* ATCC 15442, *Escherichia coli* ATCC 10536, *Enterococcus hirae* ATCC 10541, *Candida albicans* ATCC 10231, and *Aspergillus brasiliensis* ATCC 16404. First, the discs (2 × 2 cm) were sterilized with isopropanol (the concentration 70 g/100 mL) for 15 min before each assay. Suspensions of bacteria, yeasts, and fungi were diluted (ratio 1:1) with 0.03 g/100 mL bovine serum albumin to mimic relevant working conditions (as in EN 13697:2019). Then, 50 μL of the resulting inoculum (6.98 ± 0.17–7.06 ± 0.11 in the case of bacteria, 5.90 ± 0.23–6.01 ± 0.10 in the case of yeasts and fungi) were spotted into sterile discs and dried at 20 °C for 15 min. Afterwards, 100 μL of preparations 1 and 2 (P1 and P2) at a concentration of 0.6 and 60 g/100 mL diluted with distilled water as diluent according to EN 13697:2019 were spotted on the inoculated discs, followed by incubation at 20 ± 1 °C for 60 ± 10 s and 300 ± 10 s. Then, the effect of the preparations was stopped by transferring the discs into a flask with 10 mL of neutralizer. Thereafter, by shaking at 240 rpm bacterial, yeasts, and fungi cells were enumerated as described above. The stability of the preparations in a mixture with distilled water as a diluent was evaluated. No precipitate was observed during the test. The method used is intended to confirm the performance of the preparations under laboratory conditions similar to the intended use as disinfectants and antiseptics in households, food industry, and public facilities; see Table 7 (EN 13697:2019). Moreover, the evaluation of the concentration at which the plant preparations diluted in distilled water showed antimicrobial activity against the tested reference strains on the tested surfaces for 60 ± 10 s and 300 ± 10 s, at 20 ± 1 °C and under dirty conditions. Additionally, the aim of this part of the study was to indicate the concentration at which the tested preparations under the mentioned conditions were characterized by full antimicrobial activity

Reduction of the microbial number of microorganisms during the test (the log reduction (*LR*)) was calculated using the following formula and expressed as the log difference in the viable cell counts before and after treatment [64]:

(1) $$LR=\frac{N}{N_{ts}}=N_{ts},$$

where *N* is the log of the number of cells alive applied to the test surface (*N*) and *N_ts_* is the log of the number of cells alive remaining on the surface after the test.

Table 7 presents the conditions for determining surface disinfection tests of plant preparations by standard EN 13697:2019.

### 4.9. Surface Disinfection Tests of Plant Preparations by Standard EN 13697:2015

The surface disinfection tests of preparation 1 (P1) obtained from the leaves of *R. idaeus* L. and preparation 2 (P2) obtained from the leaves of *R. fruticosus* L. by standard EN 13697:2015 consisted of a dilution method of this preparations and their neutralization in which microorganisms were treated with the preparations at concentrations of 14, 21, 28, 35, 42, 49, 56, 63, and 70 g/100 mL for time 60 ± 10 s and at 20 ± 1 °C with the addition of a loading substance (bovine serum albumin). Using EN 13697:2015, the bactericidal activity was determined against undiluted preparations 1 and 2 at a concentration of 70 g/100 mL as well as against appropriately diluted preparations at concentrations of 14, 21, 28, 35, 42, 49, 56, and 63 g/100 mL in order to evaluate the bactericidal activity of a given preparation from the active concentration (63 g/100 mL) to the inactive concentration (14 g/100 mL) [65].

First, the method was validated using the solution neutralization technique (water containing sodium thiosulfate, soy lecithin, and Polysorbate 80). The neutralizer used allowed the method to be validated (Table 8). The antimicrobial activity of plant preparations was evaluated on discs against the following reference strains: *Staphylococcus aureus* ATCC 6538 and *Pseudomonas aeruginosa* ATCC 15442. First, the discs (2 × 2 cm) were sterilized with isopropanol at a concentration of 70 g/100 mL for 15 min before each assay. Suspensions of bacteria were diluted (ratio 1:1) with 0.3 g/100 mL bovine serum albumin to mimic relevant working conditions, as in EN 13697:2015. Then, 50 μL of resulting inoculum (6.79 ± 0.42 in the case of *Staphylococcus aureus* ATCC 6538 and 6.77 ± 0.41 in the case of *Pseudomonas aeruginosa* ATCC 15442) were spotted into sterile discs and dried at 20 °C for 15 min. Afterwards, 100 μL of preparations 1 and 2 diluted with sterile hard water as diluent according to EN 13697:2015 were spotted on the inoculated discs, followed by incubation at 20 ± 1 °C for 60 ± 10 s. Then, the effect of the preparations was stopped by transferring the discs into a flask with 10 mL of neutralizer and bacterial cells were enumerated in a shaker at 240 rpm. The stability of the preparations in a mixture with sterile hard water containing calcium carbonate at a concentration of 30 mg/100 mL CaCO_3_ as a diluent was evaluated. No precipitate was observed during the test. The method used was intended to confirm the performance of the preparations under laboratory conditions similar to the intended use as a disinfectant applied in the food sector, industrial and domestic settings, and public utilities for non-porous surfaces; see Table 8 (EN 13697:2015). Moreover, the determination of the concentration at which the plant preparations diluted in hard water showed bactericidal activity against tested reference strains on the tested surfaces for 60 ± 10 s at a temperature of 20 ± 1 °C and in unclean conditions was performed. In addition, the concentration at which the tested preparations under the mentioned conditions were characterized by full bactericidal effect was evaluated.

For comparison, the bactericidal activity of undiluted ethanol at a concentration of 100 g/100 mL and hard water diluted ethanol at concentrations of 80 and 90 g/100 mL, respectively, against *Staphylococcus aureus* ATCC 6538 and *Pseudomonas aeruginosa* ATCC 15442 was evaluated under the same conditions.

Table 8 presents the conditions for determining surface disinfection tests of plant preparations by standard EN 13697:2015.

### 4.10. Testing of Hand Disinfection by Means of Rubbing in Plant Preparations According to EN 1500:2013

The testing of hand disinfection by means of rubbing of preparation 1 (P1) obtained from the leaves of *R. idaeus* L. and preparation 2 (P2) obtained from the leaves of *R. fruticosus* L. was performed according to the EN 1500:2013 standard. The aim of this part of the study was to determine whether the plant preparations P1 and P2 reduce the release of transient microorganisms when applied to participants’ artificially contaminated hands. The testing of hand disinfection by the preparations according to the EN 1500:2013 standard consisted of rubbing with the preparations in a volume of 6 mL for time 60 ± 5 s at 20 ± 1 °C [66].

Validation of the method was performed using a solution neutralization technique. Depth culture on plates was used as a method for counting microorganisms. Statistical analysis of the test results was performed, assuming that the activity against *Escherichia coli* K12 NCTC 10538 of the tested preparation (P1 and P2) was lower than that of the reference preparation (PR). Calculations were performed using the Wilcoxon test, and the results obtained are shown in Table 9. According to the standard, the preparations obtained are considered effective if the results obtained meet the acceptance criteria, and statistical analysis of the data obtained proves that the tested preparation does not show lower antimicrobial activity than the reference preparation (propan-2-ol at a concentration of 60 g/100 mL) against the tested reference strain of *Escherichia coli* K12 NCTC 10538. 

Table 9 presents the conditions for determining the antimicrobial activity of the plant preparations according to the EN 1500:2013 standard.

### 4.11. Evaluation of Antimicrobial Activity of Plant Preparations against Isolated Strains of Bacteria, Yeasts, and Fungi

To determine the microbial activity against sixteen isolated strains of bacteria, four strains of yeasts, and five strains of fungi, preparation 1 (P1) obtained from the leaves of *R. idaeus* L. and preparation 2 (P2) obtained from the leaves of *R. fruticosus* L. were placed in a vacuum evaporator to evaporate the solvent, thus obtaining 4.55 g of dry raspberry leaf preparation and 4.55 g of dry blackberry leaf preparation. Next, sixteen strains of bacteria (*Enterococcus faecalis* strain 1–5, *Enterococcus faecium* strain 1–5, *Escherichia coli* strain 1–5, *Bacillus spp.*), four strains of yeasts (yeasts strain 1, 2, 5 and 6), and five strains of fungi (*Penicillium ciclopsis*, *Trichothecium roseum*, *Aspergillus fumigatus*, *Aspergillus niger*, *Mucor sp*.) were isolated from natural environments. The isolated strains were identified and cultured as pure cultures. Then, microbiological evaluation of the obtained dry raspberry and blackberry leaves preparations against the isolated microorganisms was carried out.

Additionally, microbiological evaluation of antibiotics by the antibiogram method against isolates (antibiotics Penicillin, Ampicillin, Vancomycin, Phosphomycin, Amphotericin, Nystatin, Ketoconazole) and reference strains (antibiotics Doxycycline, Ampicillin, Ciprofloxacin) was carried out for comparative purposes, and the effect of the preparations P1 and P2 on thirteen reference strains *Escherichia coli* (ATCC25922), *Enterococcus faecalis* (ATCC29212), *Klebsiella pneumoniae* (ATCC700603), *Pseudomonas aeruginosa* (ATCC27853), *Salmonella typhimurium* (ATCC14028), *Staphylococcus aureus* A (ATCC29213), *Staphylococcus aureus* B (ATCC25923), *Streptococcus pneumoniae* (ATCC49619), *Streptococcus intermedius* (ATCC29663), *Sarcina lutea* (ATCC9341), *Bacillus subtilis, Bacillus pseudomycoides*, and *Bacillis thuringiensis* was determined. For each group of microorganisms an appropriate medium was used: for bacterial cultivation, TSA (Trypticase Soy Agar) and for yeasts, (Malt Extract Agar). The inhibitory effect of the plant preparations was assessed based on the zone of inhibition of the growth of the culture. Measurements were taken every 24 h and the results after 72 h were used for final analysis.

The strains isolated from the ground environment were from the collection of the Department of Microbiology and Environmental Chemistry, Faculty of Environmental Management and Agriculture, West Pomeranian University of Technology, Szczecin. The strains were determined by classical microbiological methods.

#### 4.11.1. Methods of Application of Plant Preparations to Medium

The preparations obtained from dry raspberry and blackberry leaves (p. 4.10) were then dissolved in 5 mL of ethanol (70 g/100 mL) to obtain an ethanolic solution containing 9100 µg of dry preparations in 10 µL of ethanol. The sensitivity of the tested microorganisms on the prepared ethanolic solutions containing dry raspberry and blackberry leaves preparations was determined using the well method and disc method [67].

In the case of the well method, the appropriate medium was poured into Petri dishes; after the medium had solidified, wells with a diameter of 8 mm were cut out using a sterile plug, into which 10–40 µL of an ethanolic solution containing dry preparations was then introduced. For the disc method, 5 mm diameter paper discs were placed on the surface of the appropriate medium and 10–40 µL of an ethanolic solution containing dry preparations was applied to them. The inhibitory effect of the of the tested solutions was assessed based on the diameter of the growth inhibition zone of microorganisms. 

#### 4.11.2. Methods of Application of Antibiotics to Medium

Determination of the susceptibility of the strains to antibiotics was performed using the antibiogram method. Prepared discs soaked in antibiotics were purchased (Pol-Aura), then the tested strain was applied on the surface of the appropriate medium. In the next stage, the discs with antibiotics were placed on the medium prepared in this way measurements were made in three repetitions. The effect on particular strains was evaluated by measuring of the size of the growth inhibition zones.

### 4.12. Measurement of Lipophilicity of the Natural Preparations

To determine the lipophilicity of P1 and P2, the values of the n-octanol/water partition coefficient (P) were examined. Evaluation of the lipophilicity of the preparations involved determination of their partition coefficient between two immiscible liquids, n-octanol and water, which model the properties of cell structures well. The partition coefficient was expressed as the logarithm ratio of substance concentrations in both phases [53].

The logarithm of the partition coefficient (logP) n-octanol/water was determined by the spectrophotometric method. The analyses were performed on a Thermo Scientific GENESYS 50 apparatus. The logP was determined for the sum of compounds having absorbances in the wavelength range of 230–400 nm. The concentration ratio of compounds in P1 and P2 was determined based on mass balance and the assumption that Lambert–Beer law is satisfied in the studied range of 230–400 nm, which can be illustrated by the equation

(2) $$P=\frac{C_{o}}{C_{w}}=\frac{C^{0}-C_{w}}{C_{w}}=\frac{S^{0}-S}{S}=\frac{\int_{{\;\;\;\Lambda \;\;\;}_{1}}^{{\;\;\;\Lambda \;\;\;}_{2}}A^{0}d\;\;\;\Lambda \;\;\;-\int_{{\;\;\;\Lambda \;\;\;}_{1}}^{{\;\;\;\Lambda \;\;\;}_{2}}Ad\;\;\;\Lambda \;\;\;}{\int_{{\;\;\;\Lambda \;\;\;}_{1}}^{{\;\;\;\Lambda \;\;\;}_{2}}Ad\;\;\;\Lambda \;\;\;}$$

where *C_o_* and *C_w_* is the concentration of the sum of the compounds in the n-octanol layer and in the aqueous layer, respectively, *S* is the area under the UV-Vis spectrum, *A* is the absorbance, 0 is the superscript in the preparation before extraction, and *Λ* is the wavelength.

Next, 5 mL of n-octanol was mixed with 5 mL of water in a 1:1 ratio containing the test preparation (P1 and P2) at an amount of 50 µL. The mixture was then shaken on a shaker (TS-2 Orbital Shaker) at a constant temperature of 25 °C for an appropriate time until equilibrium was reached, with the temperature controlled by an immersion thermostat. The sums of the concentrations of compounds present in the analyzed preparations were determined by spectrophotometry at wavelengths of 230–400 nm (Figure 5). Furthermore, blanks were performed for each preparation tested under the same conditions. For this purpose, n-octanol was mixed with water in a 1:1 ratio, followed by the above procedure [53]. 

### 4.13. Statistical Analysis

The results were presented as mean ± standard deviation (SD). For microbiological analysis, one-way analysis of variance (ANOVA) was used. A cluster analysis was carried out to determine the characteristics on the tested microorganisms of the ethanolic solutions containing dry preparations of raspberry and blackberry leaves action. On this basis, the effect of different doses of ethanolic solution on the tested microorganism was evaluated. The significance of differences between individual groups was evaluated with Tukey’s test (*p* < 0.05). Moreover, the significance of differences between groups in the surface disinfection tests and hand disinfection tests was evaluated using the Wilcoxon test (*p* > 0.01). Statistical calculations were carried out using Statistica 13 PL software (StatSoft, Kraków, Poland).

All of the experiments were carried out independently in triplicate, and at least three analyses per replication were performed.

## 5. Conclusions

In this study, we have devoted particular attention to species of the genus *Rubus* because of their biological and antioxidant activity, biologically active compounds, and polyphenol content. Preparations 1 and 2 were shown to contain biologically active compounds (Table 1, Figures S1–S3) and to have antioxidant and antimicrobial activities against strains isolated from the environment as well as against reference bacteria (Table 2, Table 3, Table 4 and Table 5, Tables S1–S3, Figure 3 and Figure 4). 

In addition, the quantitative method for determining the bactericidal and fungicidal activity of chemical disinfectants and antiseptics (PN-EN 13697:2019) clearly demonstrated that the resulting preparations with reduced ethanol content exhibit bactericidal and fungicidal activity on surfaces. Moreover, testing of hand disinfection according to EN 1500:2013 using preparations obtained from the leaves of *R. idaeus* L. and *R. fruticosus* L. confirmed their antimicrobial activity against *Escherichia coli* K12 NCTC 10538. Additionally, the Office for Registration of Medicinal Products, Medical Devices, and Biocidal Products (Warsaw, Poland) has issued permits for the use of natural biocidal products (P1 and P2). 

The highest antibacterial activity (growth inhibition zone 21.7 mm in the case of P1 and growth inhibition zone 19.7 mm in the case of P2) was demonstrated against the *Streptococcus intermedius* reference strain (Table 6). *Trichothecium roseum* was sensitive to the raspberry preparation, in contrast to all antibiotics tested, which showed no activity against the isolated *Trichothecium roseum* strain (Figure 3). Moreover, the raspberry preparation had higher activity than Doxycycline against the *Klebsiella pneumoniae* strain and showed a statistically significant larger zone of growth inhibition (8.3 mm) than the tested antibiotic (5.7 mm); see Table 6.

Lipophilicity studies have shown that the studied preparations obtained from the leaves of *R. idaeus* L. and *R. fruticosus* L. are highly hydrophilic. According to the results, high polyphenolic content and other biologically active compounds can be considered one of the responsible parameters for effective the biological activity of plant preparations obtained from wild plant species of the genus Rubus. Natural preparations can act as disinfectants used in the food sector, industrial and domestic settings, and public facilities. In addition, their high antioxidant potential could allow for its application as good natural sources for use in the cosmetic and pharmaceutical industries.

## Figures and Tables

**Table 1:** The components of the preparations obtained from leaves of *Rubus idaeus* L. and *Rubus fruticosus* L. as determined by GC-MS.

Chemical Compound	Retention Time (min)	Formula	Biological Activity
2-hexenal ^1,2^	5.18	C_6_H_10_O	-
2-Heptanone ^1,2^	5.31	C_7_H_14_O	-
2-hexanol-3-methyl ^1,2^	5.41	C_7_H_16_O	-
4-heptanol-3-ethyl ^1,2^	5.56	C_9_H_20_O	-
3-hexanol-5-methyl ^1,2^	7.81	C_7_H_16_O	-
4H-pyran-4-one ^1,2^	10.48	C_6_H_8_O_4_	- [23]
5-(hydroxymethyl)furfural ^1,2^	11.58	C_6_H_6_O_3_	- [35,41]
2,4-heptadienal ^1,2^	12.98	C_7_H_10_O	antimicrobial, antifungal [42,43]
2-honanone ^1,2^	13.69	C_9_H_18_O	
Pyrogallol ^1,2^	14.54	C_6_H_6_O_3_	multi-directional biological activity [42,43,44]
2-hydroxy-5-methylbenzaldehyde ^2^	14.59	C_8_H_8_O_2_	- [23]
n-decanoic acid ^2^	16.51	C_10_H_20_O_2_	antifungal, antibacterial [42,43]
quinic acid ^2^	17.01	C_7_H_12_O_6_	antioxidant, anti-inflammatory [45,46]
dodecanoic acid ^1,2^	19.58	C_12_H_24_O_2_	antifungal, antibacterial [42,43]
hexadecanoic acid ^1,2^	20.60	C_16_H_32_O_2_	antioxidant, anti-inflammatory, antibacterial [9,47,48]
linoleic acid methyl ester ^1,2^	22.37	C_19_H_32_O_2_	antibacterial, antifungal [6,9,46,48]

^1^—preparation from leaves of *Rubus idaeus* L., ^2^— preparation from leaves of *Rubus fruticosus* L.

**Table 2:** Antioxidant activity and total polyphenol content of preparations obtained from the *R. idaeus* L. and *R. fruticosus* L. leaves.

	Antioxidant Activity:			Total Polyphenol Content:
Preparations	DPPH	ABTS	FRAP	F-C
	(mg Trolox/g Raw Material)			(mg GA/g Raw Material)
P1 *Rubus idaeus* L. *	3.38 ± 0.03 a	17.89 ± 1.07 b	6.13 ± 0.17 a	6.52 ± 0.57 ab
P2 *Rubus fruticosus* L. *	3.43 ± 0.02 ab	20.01 ± 0.20 a	7.39 ± 0.13 ab	7.55 ± 0.43 a

* Mean ± S.D. (n = 3), a,b—different letters: values differ significantly between the analyzed preparations.

**Table 3:** The results of surface disinfection tests of preparations 1 and 2 by standard EN 13697:2019.

EN 13697:2019 (Phase 2 Stage 2)							
Test Preparation	Treatment	*Staphylococcus aureus* ATCC 6538	*Pseudomonas aeruginosa* ATCC 15442	*Escherichia coli* ATCC 10536	*Enterococcus hirae* ATCC 10541	*Candida* *albicans* ATCC 10231	*Aspergillus brasiliensis* ATCC 16404
LR/% reduction P1 Concentration 0.6 g/100 mL	contact time 60 ± 10 s	1.45 ± 0.06b/>96.4	1.44 ± 0.04c/>96.3	1.42 ± 0.01a/>96.1	1.38 ± 0.11a/>95.8	0.42 ± 0.10b/>61.9	0.60 ± 0.03c/>74.8
LR/% reduction P1 Concentration 60 g/100 mL		>4.83/ >99.9	>4.81/ >99.9	>4.79/ >99.9	>4.08/ >99.9	>3.79/ >99.9	>3.67/ >99.9
LR/% reduction P1 Concentration 60 g/100 mL	contact time 300 ± 10 s	>4.83/ >99.9	>4.81/ >99.9	>4.79/ >99.9	>4.76/ >99.9	>3.79/ >99.9	>3.67/ >99.9
LR/% reduction P2 Concentration 0.6 g/100 mL	contact time 60 ± 10 s	1.42 ± 0.06b/ >96.1	1.35 ± 0.12a/ >95.5	1.38 ± 0.11c/ >95.8	1.39 ± 0.14a/ >95.9	0.62 ± 0.11d/ >76.0	0.41 ± 0.18a/ >61.0
LR/% reduction P2 Concentration 60 g/100 mL		3.90 ± 0.04a/ >99.9	>4.72/ >99.9	>4.75/ >99.9	>4.76/ >99.9	>3.69/ >99.9	>3.78/ >99.9
LR/% reduction P2 Concentration 60 g/100 mL	contact time 300 ± 10 s	>4.79/ >99.9	>4.72/ >99.9	>4.75/ >99.9	>4.76/ >99.9	>3.69/ >99.9	>3.78/ >99.9

Each value is the mean of three replications with the standard deviation in three independent experiments. Any two means in the same column followed by the same letter are not significantly (*p* > 0.01) different by Tukey’s multiple range tests. LR—log reduction, with the value calculated from Equation (1); P1—preparation 1, obtained from the leaves of *R. idaeus* L.; P2—preparation 2, obtained from the leaves of *R. fruticosus* L.; a,b,c,d—different letters mean that values differ significantly between the analyzed preparations.

**Table 4:** The results of surface disinfection tests of preparations 1 and 2 by standard EN 13697:2015.

EN 13697: 2015 (Phase 2 Stage 2)			
Test Preparation	Treatment	*Staphylococcus aureus* ATCC 6538	*Pseudomonas aeruginosa* ATCC 15442
Preparation 1 (P1)			
LR/% reduction Concentration of P1 14 g/100 mL	contact time 60 ± 10 s	1.74 ± 0.09 b/>98.1	1.73 ± 0.14 a/>98.1
LR/% reduction Concentration of P1 21 g/100 mL		1.98 ± 0.07 c/>98.9	1.83 ± 0.04 b/>98.5
LR/% reduction Concentration of P1 28 g/100 mL		2.25 ± 0.06 a/>99.4	2.21 ± 0.01 a/>99.3
LR/% reduction Concentration of P1 35 g/100 mL		3.28 ± 0.10 b/>99.9	2.47 ± 0.08 a/>99.6
LR/% reduction Concentration of P1 42 g/100 mL		4.35 ± 0.13 c/>99.9	3.70 ± 0.09 c/>99.9
LR/% reduction Concentration of P1 49 g/100 mL		4.78 ± 0.04 a/>99.9	4.75 ± 0.11 d/>99.9
LR/% reduction Concentration of P1 56 g/100 mL		>7.10/>99.9	>7.11/>99.9
LR/% reduction Concentration of P1 63 g/100 mL		>7.10/>99.9	>7.11/>99.9
LR/% reduction Concentration of P1 70 g/100 mL		>7.10/>99.9	>7.11/>99.9
Preparation 2 (P2)			
LR/% reduction Concentration of P2 14 g/100 mL	contact time 60 ± 10 s	1.84 ± 0.01 b/>98.5	1.76 ± 0.05 b/>98.2
LR/% reduction Concentration of P2 21 g/100 mL		2.19 ± 0.06 a/>99.3	1.92 ± 0.09 a/>98.7
LR/% reduction Concentration of P2 28 g/100 mL		2.31 ± 0.07 d/>99.5	2.35 ± 0.02 b/>99.5
LR/% reduction Concentration of P2 35 g/100 mL		4.46 ± 0.01 c/>99.9	4.31 ± 0.09 b/>99.9
LR/% reduction Concentration of P2 42 g/100 mL		>7.12/>99.9	>7.08/>99.9
LR/% reduction Concentration of P2 49 g/100 mL		>7.12/>99.9	>7.08/>99.9
LR/% reduction Concentration of P1 56 g/100 mL		>7.12/>99.9	>7.08/>99.9
LR/% reduction Concentration of P2 63 g/100 mL		>7.12/>99.9	>7.08/>99.9
LR/% reduction Concentration of P2 70 g/100 mL		>7.12/>99.9	>7.08/>99.9
Ethanol (E)			
LR/% reduction Concentration of E 80 g/100 mL		3.93 ± 0.05 b/>99.9	3.93 ± 0.07 c/>99.9
LR/% reduction Concentration of E 90 g/100 mL		>7.11/>99.9	>7.11/>99.9
LR/% reduction Concentration of E 100 g/100 mL		>7.11/>99.9	>7.11/>99.9

Each value is the mean of three replications with the standard deviation in three independent experiments. Any two means in the same column followed by the same letter are not significantly (*p* > 0.01) different by Tukey’s multiple range tests. LR—log reduction, with the value calculated from Equation (1); P1—preparation 1, obtained from the leaves of *R. idaeus* L.; P2—preparation 2, obtained from the leaves of *R. fruticosus* L.; E—Ethanol; a,b,c,d—different letters indicate that values differ significantly between the analyzed preparations.

**Table 5:** Statistical analysis of plant preparations P1 and P2 and the reference preparation (PR) according to EN 1500:2013 against *Escherichia coli* K12 strain NCTC 10538.

Tester Number	LR		PR-P1	LR		PR-P2
	PR	P1		PR	P2	
1	3.39 ± 0.11 a	4.31 ± 0.01 a	−0.92 ± 0.05 a	3.39 ± 0.11 d	3.24 ± 0.12 d	0.15 ± 0.14 b
2	4.45 ± 0.09 a	4.76 ± 0.01 a	−0.30 ± 0.05 b	4.45 ± 0.19 d	4.54 ± 0.09 cd	−0.09 ± 0.20 c
3	4.41 ± 0.08 ab	4.52 ± 0.03 ab	−0.12 ± 0.4 ab	4.41 ± 0.06 c	4.44 ± 0.05 c	−0.03 ± 0.08 c
4	3.69 ± 0.21 a	3.83 ± 0.06 b	−0.13 ± 0.12 ab	3.69 ± 0.11 cd	3.59 ± 0.03 c	0.11 ± 0.06 c
5	3.17 ± 0.19 a	3.68 ± 0.09 c	−0.50 ± 0.012 a	3.17 ± 0.13 cd	3.51 ± 0.01 b	−0.34 ± 0.06 bc
6	3.99 ± 0.08 ab	4.07 ± 0.21 c	−0.08 ± 0.02 ab	3.99 ± 0.14 cd	3.74 ± 0.09 bc	0.24 ± 0.21 d
7	4.91 ± 0.16 ab	4.09 ± 0.08 bc	0.82 ± 0.04 b	4.91 ± 0.10 e	5.82 ± 0.06 cd	−0.90 ± 0.12 d
8	3.14 ± 0.19 b	3.26 ± 0.03 ab	−0.12 ± 0.02 a	3.14 ± 0.23 e	3.06 ± 0 d	0.08 ± 0.04 e
9	3.48 ± 0.17 ab	4.06 ± 0.04 bc	−0.58 ± 0.05 ab	3.48 ± 0.05 e	4.04 ± 0.21 cd	−0.56 ± 0.01 e
10	3.78 ± 0.04 a	4.09 ± 0.05 bc	−0.32 ± 0.21 c	3.78 ± 0.05 de	3.64 ± 0.12 ab	0.14 ± 0.10 de
11	3.85 ± 0.09 c	4.22 ± 0.04 cd	−0.37 ± 0.20 d	3.85 ± 0.08 d	4.54 ± 0.10 d	−0.69 ± 0.05 c
12	3.93 ± 0.11 c	3.98 ± 0.03 ab	−0.04 ± 0.12 ab	3.93 ± 0.14 c	3.67 ± 0.07 c	0.27 ± 0.10 d
13	3.49 ± 0.13 d	3.99 ± 0.09 cd	−0.50 ± 0.05 ab	3.49 ± 0.19 d	3.61 ± 0.12 a	−0.13 ± 0.12 cd
14	3.56 ± 0.11 cd	3.94 ± 0.03 de	−0.38 ± 0.18 e	3.56 ± 0.12 de	3.61 ± 0.08 c	−0.05 ± 0.10 e
15	3.49 ± 0.19 e	3.91 ± 0.04 d	−0.43 ± 0.13 c	3.49 ± 0.09 c	3.74 ± 0.08 a	−0.26 ± 0.12 a
16	3.37 ± 0.12 ab	3.51 ± 0.08 e	−0.14 ± 0.10 b	3.37 ± 0.04 b	3.32 ± 0.06 b	0.05 ± 0.14 b
17	3.73 ± 0.19 bc	4.74 ± 0.08 d	−1.01 ± 0.13 de	3.73 ± 0.02 b	3.65 ± 0.05 b	0.08 ± 0.11 ab
18	3.69 ± 0.18 b	4.01 ± 0.01 de	−0.32 ± 0.15 ab	3.69 ± 0.10 bc	3.62 ± 0.09 b	0.07 ± 0.06 cd
19	3.65 ± 0.10 b	4.07 ± 0.03 de	−0.43 ± 0.10 b	3.65 ± 0.13 bc	3.60 ± 0.05 b	0.05 ± 0.07 c
20	3.70 ± 0.10 b	4.12 ± 0.05 b	−0.42 ± 0.04 ab	3.70 ± 0.21 de	3.50 ± 0.20 a	0.20 ± 0.05 d

Each value is the mean of three replications with the standard deviation in three independent experiments. The confidence level of the test was set at *p* = 0.025. The product is considered less active than the reference at a *LR* limit of 0.60, the median value of PRP1 is −0.34, and that of PRP2 is 0.05, LR—log reduction value as calculated from Equation (1); PR—reference preparation (propan-2-ol at a concentration of 60 g/100 mL); P1—preparation 1, obtained from the leaves of *R. idaeus* L.; P2—preparation 2, obtained from the leaves of *R. fruticosus* L.; a,b,c,d,e—different letters indicate that values differ significantly between the analyzed preparations.

**Table 6:** Effects of plant preparations and antibiotics on the tested bacterial reference strains. Results were from three independent experiments (n = 3). Mean (± standard deviation).

Bacterial/Symbol	*R. idaeus*	*R. fruticosus*	Doxycycline	Ampicillin	Ciprofloxacin
	The Diameter of the Growth Inhibition Zone (mm)				
*Escherichia coli* ATCC25922	8.7 ± 0.32 c	8.3 ± 0.30 c	20.0 ± 0.30 b	20.3 ± 0.30 b	25.0 ± 0.32 a
*Enterococcus faecalis* ATCC29212	15.0 ± 0.39 c	18.3 ± 0.12 c	35.0 ± 0.12 a	18.3 ± 0.12 c	24.7 ± 0.06 b
*Klebsiella pneumoniae* ATCC700603	8.3 ± 0.30 bc	6.3 ± 0.32 cd	5.7 ± 0.52 d	9.7 ± 0.58 b	19.3 ± 0.01 a
*Pseudomonas aeruginosa* ATCC27853	8.7 ± 0.50 d	6.0 ± 0.30 d	49.7 ± 1.04 a	38.3 ± 0.06 b	31.7 ± 0.58 c
*Salmonella typhimurium* ATCC14028	7.0 ± 0.55 d	6.0 ± 0.52 d	30.3 ± 0.58 b	17.7 ± 0.30 c	37.0 ± 0.12 a
*Staphylococcus aureus* (A) ATCC29213	16.7 ± 0.06 c	18.0 ± 0.12 c	21.7 ± 0.30 bc	26.7 ± 0.32 ab	28.3 ± 0.58 a
*Staphylococcus aureus* (B) ATCC25923	17.3 ± 0.01 c	15.3 ± 0.58 c	41.7 ± 1.00 a	35.3 ± 0.12 b	33.7 ± 0.50 b
*Streptococcuspneumoniae* ATCC49619	6.3 ± 0.15 d	6.0 ± 0.06 d	36.3 ± 0.30 a	20.0 ± 0.52 c	32.3 ± 0.55 b
*Streptococcus intermedius* ATCC29663	21.7 ± 0.59 c	19.7 ± 1.00 c	54.3 ± 2.00 a	35.3 ± 0.30 b	32.0 ± 0.06 b
*Sarcina lutea* ATCC9341	18.0 ± 0.58 d	14.3 ± 0.30 de	54.0 ± 0.01 a	35.0 ± 0.32 b	26.7 ± 0.12 c
*Bacillus subtilis*	14.0 ± 0.52 b	15.8 ± 0.52 b	32.3 ± 0.30 a	31.7 ± 0.30 a	34.3 ± 0.52 a
*Bacillus pseudomycoides*	15.3 ± 0.80 d	12.7 ± 0.30 d	25.3 ± 0.58 c	30.0 ± 0.30 b	37.3 ± 0.30 a
*Bacillis thuringiensis*	15. 0 ± 0.12 c	12.3 ± 0.05 c	14.3 ± 0.30 c	30.0 ± 0.30 b	34.3 ± 0.32 a

a,b,c,d—different letters indicate that values differ significantly between the analyzed bacteria.

**Table 7:** The conditions for determining surface disinfection tests of plant preparations by standard EN 13697:2019.

EN 13697:2019 (Phase 2 Stage 2)							
Test Preparation	Treatment	*Staphylococcus aureus* ATCC 6538	*Pseudomonas aeruginosa* ATCC 15442	*Escherichia coli* ATCC 10536	*Enterococcus hirae* ATCC 10541	*Candida* *albicans* ATCC 10231	*Aspergillus brasiliensis* ATCC 16404
Preparation 1 (P1)							
N		7.04 ± 0.41 b	6.99 ± 0.10 ab	6.98 ± 0.17 ab	7.06 ± 0.11 bc	5.90 ± 0.23 a	6.01 ± 0.10 ab
NT		6.94 ± 0.43 b	6.92 ± 0.31 ab	6.91 ± 0.19 c	6.86 ± 0.14 a	5.79 ± 0.18 ab	5.92 ± 0.17 ab
NC		6.95 ± 0.49 b	6.90 ± 0.21 ab	6.92 ± 0.25 bc	6.89 ± 0.07 b	5.79 ± 0.13 bc	5.91 ± 0.20 ab
NW		6.97 ± 0.37 a	6.96 ± 0.17 b	6.94 ± 0.24 c	6.90 ± 0.12 bc	5.81 ± 0.19 cd	5.94 ± 0.18 b
ND Concentration of P1 0.6 g/100 mL	contact time 60 ± 10 s	>5.52	>5.52	>5.52	>5.52	>5.22	>5.52
ND Concentration of P1 60 g/100 mL		<2.15	<2.15	<2.15	<2.15	<20.09	<2.15
ND Concentration of P1 60 g/100 mL	contact time 300 ± 10 s	<2.15	<2.15	<2.15	<2.15	<2.15	<2.15
Preparation 2 (P2)							
N		6.99 ± 0.21 a	6.95 ± 0.19 c	6.97 ± 0.32 a	7.02 ± 0.19 a	5.89 ± 0.08 d	5.98 ± 0.23 b
NT		6.91 ± 0.15 d	6.83 ± 0.04 a	6.86 ± 0.31 b	6.89 ± 0.14 a	5.80 ± 0.03 e	5.90 ± 0.23 b
NC		6.90 ± 0.12 cd	6.84 ± 0.05 b	6.88 ± 0.35 b	6.87 ± 0.09 b	5.77 ± 0.04 d	5.90 ± 0.27 a
NW		6.94 ± 0.11 bc	6.87 ± 0.05 c	6.90 ± 0.35 b	6.91 ± 0.23 bc	5.83 ± 0.32 a	5.92 ± 0.32 b
ND Concentration of P2 0.6 g/100 mL	contact time 60 ± 10 s	>5.52	>5.52	>5.52	>5.52	>5.22	>5.22
ND Concentration of P2 60 g/100 mL		3.04 ± 0.12 a	<2.15	<2.15	<2.15	<2.15	<2.15
ND Concentration of P2 60 g/100 mL	contact time 300 ± 10 s	<2.15	<2.15	<2.15	<2.15	<2.15	<2.15

Each value is the mean of three replications with the standard deviation in three independent experiments. Any two means in the same column followed by the same letter are not significantly (*p* >0.01) different by Tukey’s multiple range tests; a,b,c,d—different letters indicate that values differ significantly between the analyzed microorganisms. NT—log of the amount of microorganisms/mL that was applied to the test glass surface during the neutralizer toxicity test; N—log of the amount of microorganisms/mL that was applied to the test glass surface during the EN 13697:2019 test; NC—log of the amount of microorganisms/mL that was applied to the test glass surface during the validation test; NW—log of the amount of microorganisms/mL that was applied to the test glass surface during the control tests with distilled water; ND—log of the amount of microorganisms/mL that was applied to the test glass surface during antimicrobial testing.

**Table 8:** The conditions for determining surface disinfection tests of plant preparations by standard EN 13697:2015.

EN 13697:2015 (Phase 2 Stage 2)			
Test Preparation	Treatment	*Staphylococcus aureus* ATCC 6538	*Pseudomonas aeruginosa* ATCC 15442
Preparation 1 (P1)			
N		6.79 ± 0.42 b	6.77 ± 0.41 b
NT		7.17 ± 0.43 b	7.17 ± 0.43 b
NC		7.20 ± 0.48 b	7.19 ± 0.49 b
NW		7.20 ± 0.36 a	7.21 ± 0.37 a
ND Concentration of P1 14 g/100 mL	contact time 60 ± 10 s	5.46 ± 0.37 a	5.48 ± 0.23 b
ND Concentration of P1 21 g/100 mL		5.22 ± 0.09 b	5.38 ± 0.19 a
ND Concentration of P1 28 g/100 mL		4.95 ± 0.11 a	5.00 ± 0.15 b
ND Concentration of P1 35 g/100 mL		3.92 ± 0.11 a	4.74 ± 0.37 a
ND Concentration of P1 42 g/100 mL		2.85 ± 0.19 a	3.51 ± 0.33 a
ND Concentration of P1 49 g/100 mL		2.33 ± 0.10 a	2.46 ± 0.39 a
ND Concentration of P1 56 g/100 mL		<0.10	<0.10
ND Concentration of P1 63 g/100 mL		<0.10	<0.10
ND Concentration of P1 70 g/100 mL		<0.10	<0.10
Preparation 2 (P2)			
N		6.84 ± 0.41 b	6.80 ± 0.10 ab
NT		7.22 ± 0.43 b	7.16 ± 0.31 ab
NC		7.21 ± 0.49 b	7.17 ± 0.21 ab
NW		7.22 ± 0.37 a	7.18 ± 0.17 b
ND Concentration of P2 14 g/100 mL	contact time 60 ± 10 s	5.38 ± 0.14 a	5.42 ± 0.33 a
ND Concentration of P2 21 g/100 mL		5.03 ± 0.37 a	5.26 ± 0.33 a
ND Concentration of P2 28 g/100 mL		4.91 ± 0.31 a	4.83 ± 0.35 a
ND Concentration of P2 35 g/100 mL		2.76 ± 0.39 a	2.87 ± 0.37 a
ND Concentration of P2 42 g/100 mL		<0.10	<0.10
ND Concentration of P2 49 g/100 mL		<0.10	<0.10
ND Concentration of P1 56 g/100 mL		<0.10	<0.10
ND Concentration of P2 63 g/100 mL		<0.10	<0.10
ND Concentration of P2 70 g/100 mL		<0.10	<0.10
Ethanol (E)			
N		6.76 ± 0.37 a	6.77 ± 0.10 a
NT		7.19 ± 0.31 a	7.18 ± 0.12 a
NC		7.21 ± 0.34 a	7.20 ± 0.42 a
NW		7.21 ± 0.38 a	7.21 ± 0.37 a
ND Concentration of E 80 g/100 mL		3.29 ± 0.29 a	3.29 ± 0.30 a
ND Concentration of E 90 g/100 mL		<0.10	<0.10
ND Concentration of E 100 g/100 mL		<0.10	<0.10

Each value is the mean of three replicates with the standard deviation in three independent experiments. Any two means in the same column followed by the same letter are not significantly (*p* > 0.01) different by Tukey’s multiple range tests. Medium used: Trypticasein Soy LAB-Agar (TSA); neutralizer used: solution of Polysorbate 80 (3.0 g/100 mL), sodium thiosulphate (0.3 g/100 mL), and soy lecithin (0.3 g/100 mL); incubation conditions: 24 h at 37 ± 1 °C; loading substance: bovine serum albumin (0.3 g/100 mL); diluent used during the test: sterile hard water 30 mg/100 g CaCO_3_; test method and its validation: neutralization method for solutions; test temperature: 20 ± 1 °C; method of microbial counting: deep well plate inoculation; stability of the preparation/diluent mixture: no precipitate formed during the test. NT—log of the amount of microorganisms/mL applied to the test glass surface during neutralizer toxicity test; N—log of the amount of microorganisms/mL applied to the test glass surface during EN 13697:2015 tests; NC—log of the amount of microorganisms/mL applied to the test glass surface during validation test; NW—log of the amount of microorganisms/mL applied to the test glass surface during control tests with hard water; ND—log of the amount of microorganisms/mL applied to the test glass surface during antimicrobial testing.

**Table 9:** Conditions for determining the antimicrobial activity of plant preparations according to the EN 1500:2013 standard.

EN 1500:2013		
Test Preparation	*Escherichia coli* K12 NCTC 10538	
	Preparation 1 (P1)	Preparation 2 (P2)
N	8.63 ± 0.11 a	8.63 ± 0.17 a
Nvb	97 × 10^4^	98 × 10^4^
B	88 ± 0.23 b	93 ± 0.43 b
Nv	750 ± 0.31 a	860 ± 0.21 a
C	71 ± 0.28 a	81 ± 0.13 a

Each value is the mean of three replications with the standard deviation in three independent experiments. Any two means in the same column followed by the same letter are not significantly (*p* > 0.01) different by Tukey’s multiple range tests. Medium used: Trypticasein Soy LAB-Agar (TSA) determination of N, B and C parameters and Trypticasein Selective Soy LA B Agar (TSSA) determination of the number of microorganisms in the tested preparations; neutralizer used: solution of Polysorbate 80 (3.0 g/100 mL), sodium thiosulphate (1.0 g/100 mL), and soy lecithin (0.3 g/100 mL); incubation conditions: 24 h at 37 ± 1 °C; loading substance: bovine serum albumin (0.03 g/100 mL); diluent used during test: distilled water; test method and its validation: neutralization method for solutions; test temperature: 20 ± 1 °; method of microbial counting: deep well plate inoculation; rubbing-in method used: according of EN 1500:2013; reference biocide: propan-2-ol concentration 60 g/100 mL; amount of preparation used in the test: 6 mL; contact time of the product with the bacterial suspension: 60 ± 5 s. N—log of the amount of microorganisms/mL in the EN 1500:2013 test mixture; Nvb—the amount of microorganisms/mL used during the neutralizer toxicity test; Nv—amount of microorganisms/mL used during the validation test.

## Data Availability

The data presented in this study are available in this article.

## Supplementary Materials

The following supporting information can be downloaded at: https://www.mdpi.com/article/10.3390/molecules27175486/s1. Supplementary data: GC-MS chromatogram of preparation 1 (P1) obtained from leaves of *Rubus idaeus* L. (Figure S1), GC-MS chromatogram of preparation 2 (P2) obtained from leaves of *Rubus fruticosus* L. (Figure S2), The structures of the compounds identified in the tested preparations obtained from the leaves of *R. idaeus* L. and *R. fruticosus* L. (Figure S3), The sorted values for the statistical analysis of plant preparations P1 and P2 and the reference preparation (PR), according to EN 1500:2013 against *Escherichia coli* K12 strain NCTC 10538 (Table S1), The results of disinfection tests of plant preparations according to standard EN 13697:2019 (Table S2), The results of disinfection tests of plant preparations according to standard EN 13697:2015 (Table S3).
