# Supplementary material for: Biologically Active Preparations from the Leaves of Wild Plant Species of the Genus Rubus
Source: Molecules. 2022 Aug 26;27(17):5486. doi: 10.3390/molecules27175486 (PMC9457741; doi:10.3390/molecules27175486)
Supplement: Supplementary file 1 [file molecules-27-05486-s001.zip › molecules-1828731-supplementary.pdf]

molecules

MDPI

Supplementary

Biologically Active Preparations from the Leaves of Wild Plant Species of the Genus *Rubus*.

Łukasz Kucharski [1,*], Krystyna Cybulska [2], Edyta Kucharska [3,*], Anna Nowak [1], Robert Pełech [3] and Adam Klimowicz [1]

[1] Department of Cosmetic and Pharmaceutical Chemistry, Pomeranian Medical University in Szczecin, PL-70111 Szczecin, Poland; lukasz.kucharski@pum.edu.pl (Ł.K.); anowak@pum.edu.pl (A.N.); adam.klimowicz@pum.edu.pl (A.K.)

[2] Department of Microbiology and Environmental Chemistry, Faculty of Environmental Management and Agriculture, West Pomeranian University of Technology, Szczecin, PL-71434 Szczecin, Poland; Krystyna.Cybulska@zut.edu.pl (K.C.)

[3] Faculty of Chemical Technology and Engineering, Department of Chemical Organic Technology and Polymeric Materials, West Pomeranian University of Technology, Szczecin, PL-70322 Szczecin, Poland; edyta.kucharska@zut.edu.pl (E.K.), rpelech@zut.edu.pl (R.P.)

* Correspondence: lukasz.kucharski@pum.edu.pl (Ł.K.); Tel.: +48-660-476-340, edyta.kucharska@zut.edu.pl (E.K.); Tel.: +48-888-615-273

Citation: Lastname, F.; Lastname, F.; Lastname, F. Title. *Molecules* **2022**, *11*, x. https://doi.org/10.3390/xxxxx

Academic Editor: Firstname Lastname

Received: date
Accepted: date
Published: date

Publisher's Note: MDPI stays neutral with regard to jurisdictional claims in published maps and institutional affiliations.

Copyright: © 2021 by the authors. Submitted for possible open access publication under the terms and conditions of the Creative Commons Attribution (CC BY) license (https://creativecommons.org/licenses/by/4.0/).

Figure 1S presents the GC-MS chromatogram of the preparation obtained from leaves of the *Rubus idaeus* L.

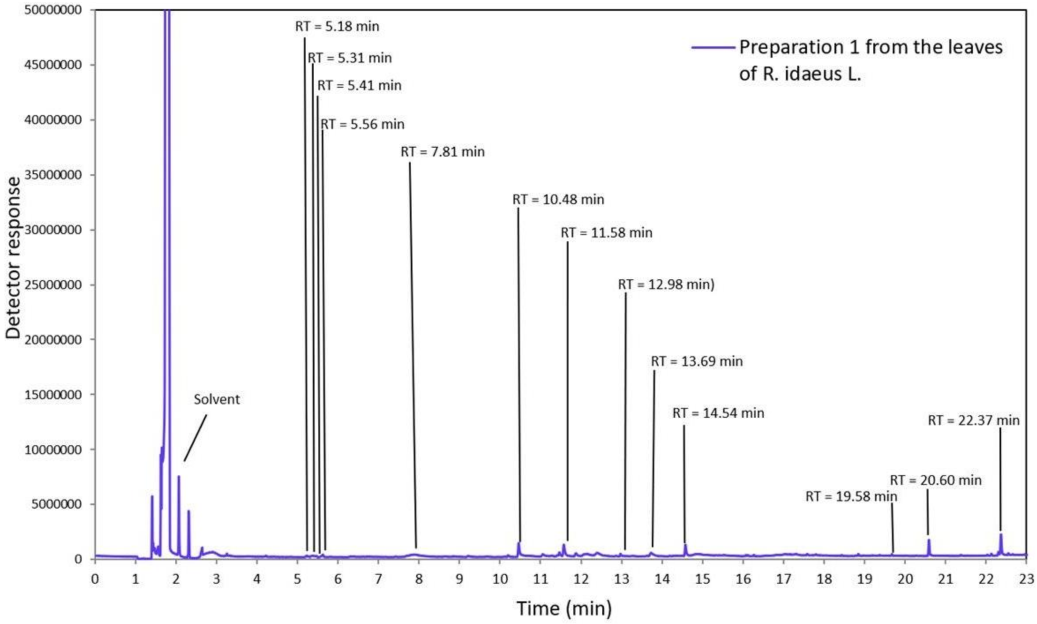

Figure 1S. GC-MS chromatogram of the preparation 1 (P1) obtained from leaves of the *Rubus idaeus* L. (2-Hexenal RT= 5.18 min, 2-Heptanone RT= min 5.31, 2-Hexanol-3-methyl RT= 5.41 min, 4-Heptanol-3-ethyl RT= 5.56 min, 3-Hexanol-5-methyl RT= 7.81 min, 4-H-pyran-4-one RT= 10.48 min, 5-(Hydroxymethyl)furfural RT= 11.58 min, 2,4-Heptadienal RT= 12.98 min, 2-Nonanone RT= 13.69 min, Pyrogallol RT= 14.54 min, Dodecanoic acid RT = 19.58 min, Hexadecanoic acid RT= 20.60 min, Linoleic acid methyl ester RT= 22.37 min).

Figure 2S presents the GC-MS chromatogram of the preparation obtained from leaves of the *Rubus fruticosus* L.

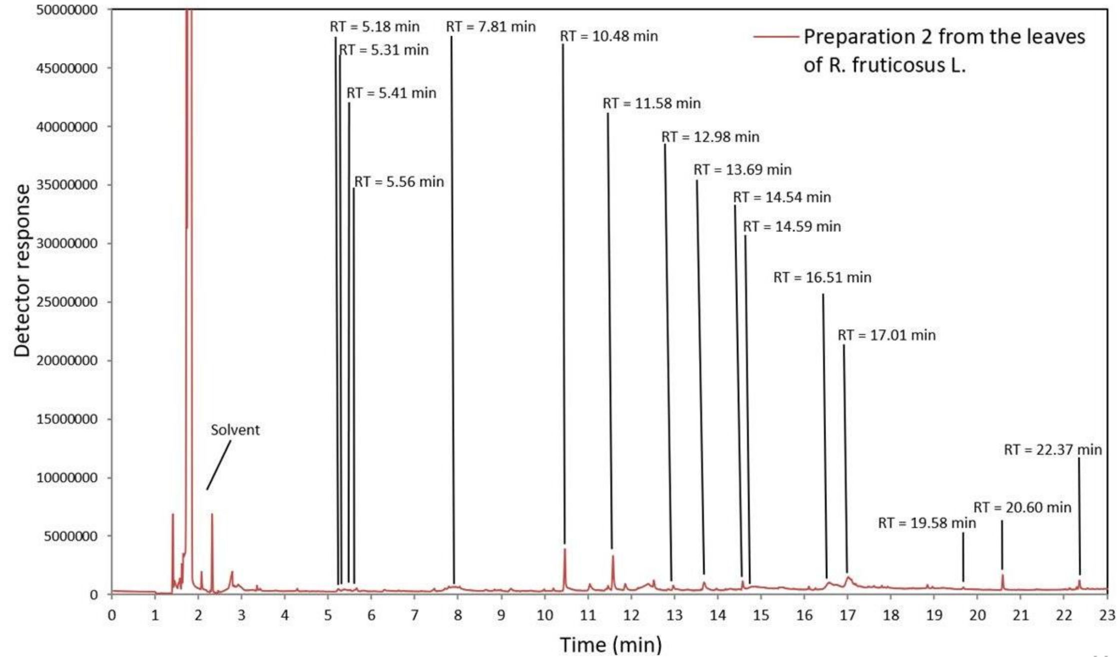

Figure 2S. GC-MS chromatogram of the preparation 2 (P2) obtained from leaves of the *Rubus fruticosus* L. (2-Hexenal RT= 5.18 min, 2-Heptanone RT= min 5.31, 2-Hexanol-3-methyl RT= 5.41 min, 4-Heptanol-3-ethyl RT= 5.56 min, 3-Hexanol-5-methyl RT= 7.81 min, 4-H-pyran-4-one RT= 10.48 min, 5-(Hydroxymethyl)furfural RT= 11.58 min, 2,4-Heptadienal RT= 12.98 min, 2-Nonanone RT= 13.69 min, Pyrogallol RT= 14.54 min, 2-Hydroxy-5-methylbenzaldehyde RT= 14.59 min, n-Decanoic acid RT= 16.51 min, Quinic acid RT= 17.01 min, Dodecanoic acid RT = 19.58 min, Hexadecanoic acid RT= 20.60 min, Linoleic acid methyl ester RT= 22.37 min).

Figure 3S shows the structures of the compounds identified in the tested preparations obtained of the leaves of *R. idaeus* L. (P1) and *R. fruticosus* L. (P2).

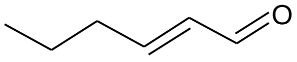

2-Hexenal RT= 5.18 min (present in preparations 1 and 2)

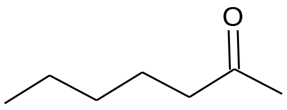

2-Heptanone RT= min 5.31 (present in preparations 1 and 2)

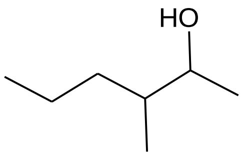

2-Hexanol-3-methyl RT= 5.41 min (present in preparations 1 and 2)

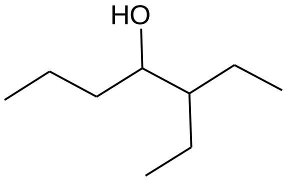

4-Heptanol-3-ethyl RT= 5.56 min (present in preparations 1 and 2)

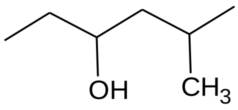

3-Hexanol-5-methyl RT= 7.81 min (present in preparations 1 and 2)

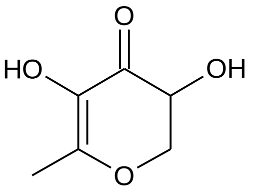

4-H-pyran-4-one RT= 10.48 min (present in preparations 1 and 2)

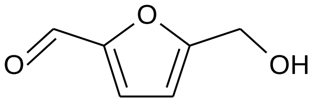

5-(Hydroxymethyl)furfural RT= 11.58 min (present in preparations 1 and 2)

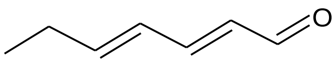

2,4-Heptadienal RT= 12.98 min (present in preparations 1 and 2)

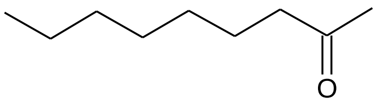

2-Nonanone RT= 13.69 min (present in preparations 1 and 2)

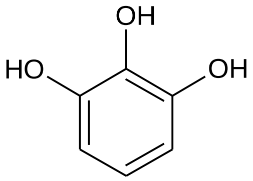

Pyrogallol RT= 14.54 min (present in preparations 1 and 2)

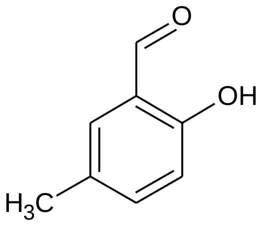

2-Hydroxy-5-methylbenzaldehyde RT= 14.59 min (present only in preparation 2)

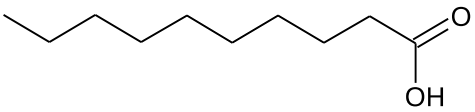

n-Decanoic acid RT= 16.51 min (present only in preparation 2)

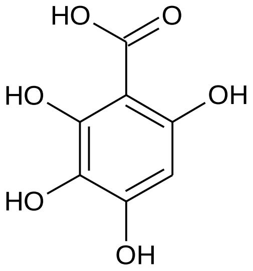

Quinic acid RT= 17.01 min (present only in preparation 2)

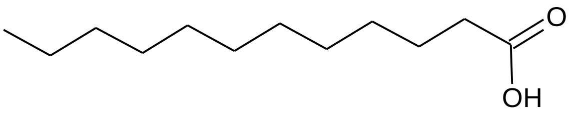

Dodecanoic acid RT = 19.58 min (present in preparations 1 and 2)

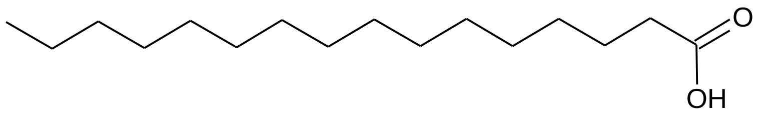

Hexadecanoic acid RT= 20.60 min (present in preparations 1 and 2)

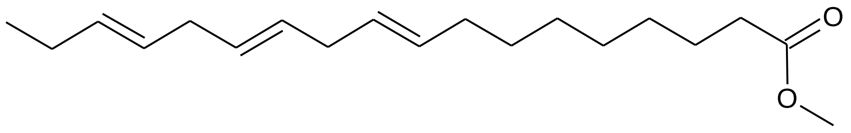

Linoleic acid methyl ester RT= 22.37 min (present in preparations 1 and 2)

Figure 3S. The structures of the compounds identified in the tested preparations obtained of the leaves of *R. idaeus* L. and *R. fruticosus* L.

Table 1S present the sorted values for the statistical analysis of plant preparations P1 and P2, and the reference preparation (PR), according to EN 1500:2013 and against *Escherichia coli* K12 strain NCTC 10538.

Table 1S. Statistical analysis of plant preparations P1 and P2, and the reference preparation (PR), according to EN 1500:2013 and against *Escherichia coli* K12 strain NCTC 10538 - sorted results and calculation results for statistical test.

PR-P1	0.82	-0.04	-0.08	-0.12	-0.12	-0.13	-0.14	-0.30	-0.32	-0.32
0.82	0.82									
-0.04	0,39	-0.04								
-0.08	0.37	-0.06	-0.08							
-0.12	0.35	-0.08	-0.10	-0.12						
-0.12	0.35	-0.08	-0.10	-0.12	-0.12					
-0.13	0.35	-0.09	-0.11	-0.13	-0.13	-0.13				
-0.14	0.34	-0.09	-0.11	-0.13	-0.13	-0.14	-0.14			
-0.30	0.26	-0.17	-0.19	-0.21	-0.21	-0.22	-0.22	-0.30		
-0.32	0.25	-0.18	-0.20	-0.22	-0.22	-0.22	-0.23	-0.31	-0.32	
-0.32	0.25	-0.18	-0.20	-0.22	-0.22	-0.23	-0.23	-0.31	-0.32	-0.32
-0.37	0.23	-0.20	-0.22	-0.24	-0.24	-0.25	-0.25	-0.33	-0.34	-0.34
-0.38	0.22	-0.21	-0.23	-0.25	-0.25	-0.26	-0.26	-0.34		
-0.42	0.20	-0.23	-0.25	-0.27	-0.27	-0.28	-0.28			
-0.43	0.20	-0.23	-0.25	-0.27	-0.27	-0.28	-0.28			
-0.43	0.20	-0.24	-0.26	-0.27	-0.27	-0.28	-0.28			
-0.50	0.16	-0.27	-0.29	-0.31	-0.31	-0.32	-0.32			
-0.50	0.16	-0.27	-0.29	-0.31	-0.31	-0.32	-0.32			
-0.58	0.12	-0.31	-0.33							
-0.92	-0.05									
-1.01	-0.09									
PR-P2	0.27	0.24	0.20	0.15	0.14	0.11	0.08	0.08	0.07	0.05
0.27	0.27									
0.24	0.25	0.24								
0.20	0.23	0.22	0.20							
0.15	0.21	0.20	0.18	0.15						

0.14	0.20	0.19	0.17	0.15	0.14					
0.11	0.19	0.18	0.15	0.13	0.12	0.11				
0.08	0.17	0.16	0.14	0.12	0.11	0.10	0.08			
0.08	0.17	0.16	0.14	0.11	0.11	0.09	0.08	0.08		
0.07	0.17	0.15	0.13	0.11	0.10	0.09	0.07	0.07	0.07	
0.05	0.16	0.15	0.12	0.10	0.10	0.08	0.07	0.06	0.06	0.05
0.05	0.16	0.14	0.12	0.10	0.09	0.08	0.06	0.06	0.06	0.05
-0.03	0.12	0.11	0.08	0.06	0.06					
-0.05	0.11	0.09	0.07	0.05						
-0.09	0.09	0.08	0.06							
-0.13	0.07	0.06								
-0.26										
-0.34										
-0.56										
-0.69										
-0.90										

PR - reference preparation (propan-2-ol at a concentration of 60 g/100 mL),
P1 - preparation 1 obtained of the leaves of *R. idaeus* L.,
P2 - preparation 2 obtained of the leaves of *R. fruticosus* L.

Table 2S. The results of disinfection tests of plant preparations by standard EN 13697:2019.

**EN 13697:2019 (phase 2 stage 2)**							
**Test preparation**	**Treatment**	****Staphylococcus aureus* ATCC 6538**	****Pseudomonas aeruginosa* ATCC 15442**	****Escherichia coli* ATCC 10536**	****Enterococcus hirae* ATCC 10541**	*****Candida albicans* ATCC 10231**	*****Aspergillus brasiliensis* ATCC 16404**
Preparation 1 (P1)							
Concentration of P1 0.6 g/100 mL	contact time 60 ± 10 s	10^{-0}:>330<br>10^{-1}:>330<br>10^{-2}:>330<br>Nts:>330<br>R: <1.45	10^{-0}:>330<br>10^{-1}:>330<br>10^{-2}:>330<br>Nts:>330<br>R: <1.44	10^{-0}:>330<br>10^{-1}:>330<br>10^{-2}:>330<br>Nts:>330<br>R: <1.42	10^{-0}:>330<br>10^{-1}:>330<br>10^{-2}:>330<br>Nts:>330<br>R: <1.38	10^{-0}:>330<br>10^{-1}:>330<br>10^{-2}:>330<br>Nts:>330<br>R: <0.42	10^{-0}:>165<br>10^{-1}:>165<br>10^{-2}:>165<br>Nts:>165<br>R: <0.60
Concentration of P1 60 g/100 mL		10^{-0}:<14<br>10^{-1}:<14<br>10^{-2}:<14<br>Nts:>0<br>R: >4.83	10^{-0}:<14<br>10^{-1}:<14<br>10^{-2}:<14<br>Nts:>0<br>R: >4.81	10^{-0}:<14<br>10^{-1}:<14<br>10^{-2}:<14<br>Nts:>0<br>R: >4.79	10^{-0}:67±0.07<br>10^{-1}:<14<br>10^{-2}:<14<br>Nts:>0<br>R: >4.08	10^{-0}:<14<br>10^{-1}:<14<br>10^{-2}:<14<br>Nts:>0<br>R: >3.79	10^{-0}:<14<br>10^{-1}:<14<br>10^{-2}:<14<br>Nts:>0<br>R: >3.67
Concentration of P1 60 g/100 mL	contact time 300 ± 10 s	10^{-0}:<14<br>10^{-1}:<14<br>10^{-2}:<14<br>Nts:>0<br>R: >4.83	10^{-0}:<14<br>10^{-1}:<14<br>10^{-2}:<14<br>Nts:>0<br>R: >4.81	10^{-0}:<14<br>10^{-1}:<14<br>10^{-2}:<14<br>Nts:>0<br>R: >4.79	10^{-0}:<14<br>10^{-1}:<14<br>10^{-2}:<14<br>Nts:>0<br>R: >4.76	10^{-0}:<14<br>10^{-1}:<14<br>10^{-2}:<14<br>Nts:>0<br>R: >3.79	10^{-0}:<14<br>10^{-1}:<14<br>10^{-2}:<14<br>Nts:>0<br>R: >3.67
Preparation 2 (P2)							
	contact time 60 ± 10 s	10^{-0}:>330<br>10^{-1}:>330<br>10^{-2}:>330	10^{-0}:>330<br>10^{-1}:>330<br>10^{-2}:>330	10^{-0}:>330<br>10^{-1}:>330<br>10^{-2}:>330	10^{-0}:>330<br>10^{-1}:>330<br>10^{-2}:>330	10^{-0}:>330<br>10^{-1}:>330<br>10^{-2}:>330	10^{-0}:>165<br>10^{-1}:>165<br>10^{-2}:>165

Concentration of P2 0.6 g/100 mL		Nts:>330<br>R: <1.42	Nts:>330<br>R: <1.35	Nts:>330<br>R: <1.38	Nts:>330<br>R: <1.39	Nts:>330<br>R: <0.41	Nts:>165<br>R: <0.62
Concentration of P2 60 g/100 mL		10^{-0}:110±0.03a<br>10^{-1}:<14<br>10^{-2}:<14<br>Nts:>40<br>R: >3.90	10^{-0}:<14<br>10^{-1}:<14<br>10^{-2}:<14<br>Nts:>0<br>R: >4.72	10^{-0}:<14<br>10^{-1}:<14<br>10^{-2}:<14<br>Nts:>0<br>R: >4.75	10^{-0}:<14<br>10^{-1}:<14<br>10^{-2}:<14<br>Nts:>0<br>R: >4.76	10^{-0}:<14<br>10^{-1}:<14<br>10^{-2}:<14<br>Nts:>0<br>R: >3.78	10^{-0}:<14<br>10^{-1}:<14<br>10^{-2}:<14<br>Nts:>0<br>R: >3.69
Concentration of P2 60 g/100 mL	contact time 300 ± 10 s	10^{-0}:<14<br>10^{-1}:<14<br>10^{-2}:<14<br>Nts:>0<br>R: >4.79	10^{-0}:<14<br>10^{-1}:<14<br>10^{-2}:<14<br>Nts:>0<br>R: >4.72	10^{-0}:<14<br>10^{-1}:<14<br>10^{-2}:<14<br>Nts:>0<br>R: >4.75	10^{-0}:<14<br>10^{-1}:<14<br>10^{-2}:<14<br>Nts:>0<br>R: >4.76	10^{-0}:<14<br>10^{-1}:<14<br>10^{-2}:<14<br>Nts:>0<br>R: >3.78	10^{-0}:<14<br>10^{-1}:<14<br>10^{-2}:<14<br>Nts:>0<br>R: >3.69

Each value is the mean of three replicates with the standard deviation in three independent experiments. Any two means in the same column followed by the same letter are not significantly (P >0.01) different by Tukey's multiple range tests,

* medium used: Trypticasein Soy LAB-Agar (TSA), neutralizer used: solution of Polysorbate 80 (3.0 g/100 mL), sodium thiosulphate (1.0g/100 mL) and soy lecithin (0.3g/100 mL), incubation conditions: 24h at 37 ± 1 °C, loading substance: bovine serum albumin (0.03g/100 mL), the diluent used during the test: distilled water, test method and its validation: neutralization method for solutions, test temperature: 20 ± 1 °C, method of microbial counting: deepwell plate inoculation, stability of the preparation/diluent mixture: no precipitate formed during the test,

** medium used: Malt-extract Agar (MEA), neutralizer used: solution of Polysorbate 80 (3.0 g/100 mL), sodium thiosulphate (1.0 g/100 mL) and soy lecithin (0.3 g/100 mL), incubation conditions: 48h at 30 ± 1 °C for yeast and 120h at 30 ± 1 °C for fungal, loading substance: bovine serum albumin (0.03g/100 mL), the diluent used during the test: distilled water, test method and its validation: neutralization method for solutions, test temperature: 20 ± 1 °C, method of microbial counting: deepwell plate inoculation, stability of the preparation/diluent mixture: no precipitate formed during the test,

Nts - the number of units remaining after the test is performed,

R - reduction in the number of microorganisms during the test,

[a] - different letters: values differ significantly between the analyzed preparations.

Table 3S. The results of disinfection tests of plant preparations by standard EN 13697:2015.

EN 13697:2015 (phase 2 stage 2)			
**Test preparation**	**Treatment**	***Staphylococcus aureus* ATCC 6538**	***Pseudomonas aeruginosa* ATCC 15442**
Preparation 1 (P1)			
Concentration of P1 14 g/100 mL	contact time 60 ± 10 s	10^{-0} :>330<br>10^{-1} :>330<br>10^{-2} :289±0.09a<br>Nts :>100<br>R: 1.74±0.01ab	10^{-0} :>330<br>10^{-1} :>330<br>10^{-2} :151±0.01a<br>Nts :100±0.01b<br>R: 1.73±0.02ab
Concentration of P1 21 g/100 mL		10^{-0} :>330<br>10^{-1} :270±0.11ab<br>10^{-2} :166.5±0.03a<br>Nts :100±0.02a<br>R: 1.98±0.04a	10^{-0} :>330<br>10^{-1} :316.5±0.10ab<br>10^{-2} :237.5±0.05a<br>Nts :100±0.06a<br>R: 1.83±0.02a
		10^{-0} :254±0.03ab	10^{-0} :271.5±0.03ab

Concentration of P1 28 g/ 100 mL		10^{-1} :163.5±0.01ab<br>10^{-2} :94.5±0.04a<br>Nts :>100<br>R: 2.25±0.01a	10^{-1} :181±0.07ab<br>10^{-2} :99.5±0.07a<br>Nts :>100<br>R: 2.21±0.01a
Concentration of P1 35 g/100 mL		10^{-0} :191±0.03a<br>10^{-1} :83.5±0.04a<br>10^{-2} :31±0.02ab<br>Nts :88±0.06ab<br>R: 3.28±0.01ab	10^{-0} :187.5±0.05a<br>10^{-1} :102.5±0.01a<br>10^{-2} :55±0.01ab<br>Nts :90±0.06ab<br>R: 2.47±0.03ab
Concentration of P1 42 g/100 mL		10^{-0} :70±0.01a<br>10^{-1} :25.5±0.03ab<br>10^{-2} :1.5±0.04b<br>Nts :22±0.09b<br>R: 4.35±0.02b	10^{-0} :81±0.08a<br>10^{-1} :32.5±0.04ab<br>10^{-2} :7.5±0.09b<br>Nts :30±0.01b<br>R: 3.70±0.03b
Concentration of P1 49 g/100 mL		10^{-0} :21.5±0.03ab<br>10^{-1} :2.5±0.02ab<br>10^{-2} :0±0.00a<br>Nts :0±0.00a<br>R: 4.87±0.03	10^{-0} :28.5±0.04ab<br>10^{-1} :10±0.01ab<br>10^{-2} :0±0.00a<br>Nts :0±0.00a<br>R: 4.75±0.01
Concentration of P1 56 g/100 mL		10^{-0} :0±0.00a<br>10^{-1} :0±0.00a<br>10^{-2} :0±0.00a<br>Nts :0±0.00a<br>R: >7.10	10^{-0} :0±0.00a<br>10^{-1} :0±0.00a<br>10^{-2} :0±0.00a<br>Nts :0±0.00a<br>R: >7.11
Concentration of P1 63 g/100 mL		10^{-0} :0±0.00a<br>10^{-1} :0±0.00a<br>10^{-2} :0±0.00a<br>Nts :0±0.00a<br>R: >7.10	10^{-0} :0±0.00a<br>10^{-1} :0±0.00a<br>10^{-2} :0±0.00a<br>Nts :0±0.00a<br>R: >7.11
Concentration of P1 70 g/100 mL		10^{-0} :0±0.00a<br>10^{-1} :0±0.00a<br>10^{-2} :0±0.00a<br>Nts :0±0.00a<br>R: >7.10	10^{-0} :0±0.00a<br>10^{-1} :0±0.00a<br>10^{-2} :0±0.00a<br>Nts :0±0.00a<br>R: >7.11
Preparation 2 (P2)			
Concentration of P2 14 g/100 mL	contact time 60 ± 10 s	10^{-0} :>330<br>10^{-1} :>330<br>10^{-2} :241.5±0.06a<br>Nts :>100<br>R: 1.84±0.02ab	10^{-0} : >330<br>10^{-1} : >330<br>10^{-2} :263.5±0.01ab<br>Nts :100±0.07a<br>R: 1.76±0.02ab
Concentration of P2 21 g/100 mL		10^{-0} :>330<br>10^{-1} :219.5±0.11a<br>10^{-2} :106.5±0.03ab	10^{-0} : >330<br>10^{-1} :243.5±0.08b<br>10^{-2} :180±0.09a

	Nts :100±0.02ab<br>R: 2.19±0.01a	Nts :100±0.04ab<br>R: 1.92±0.00a
Concentration of P2 28 g/100 mL	10^{-0} :226.5±0.01a<br>10^{-1} :123±0.03ab<br>10^{-2} :80.5±0.02sb<br>Nts :100±0.01a<br>R: 2.31±0.010b	10^{-0} :248.5±0.08a<br>10^{-1} :140±0.04b<br>10^{-2} :68±0.09b<br>Nts :100±0.03b<br>R: 2.35±0.00a
Concentration of P2 35 g/100 mL	10^{-0} :58±0.04a<br>10^{-1} :5.5±0.09b<br>10^{-2} :0±0.11a<br>Nts :0±0.09b<br>R: 4.46±0.02a	10^{-0} :74±0.08a<br>10^{-1} 28.5±0.06ab<br>10^{-2} :0±0.00ab<br>Nts :30±0.09ab<br>R: 4.31±0.03a
Concentration of P2 42 g/100 mL	10^{-0} :0±0.00a<br>10^{-1} :0±0.00a<br>10^{-2} :0±0.00a<br>Nts :0±0.00a<br>R: >7.12	10^{-0} :0±0.00a<br>10^{-1} :0±0.00a<br>10^{-2} :0±0.00a<br>Nts :0±0.00a<br>R: >7.08
Concentration of P2 49 g/100 mL	10^{-0} :0±0.00a<br>10^{-1} :0±0.00a<br>10^{-2} :0±0.00a<br>Nts :0±0.00a<br>R: >7.12	10^{-0} :0±0.00a<br>10^{-1} :0±0.00a<br>10^{-2} :0±0.00a<br>Nts :0±0.00a<br>R: >7.08
Concentration of P1 56 g/100 mL	10^{-0} :0±0.00a<br>10^{-1} :0±0.00a<br>10^{-2} :0±0.00a<br>Nts :0±0.00a<br>R: >7.10	10^{-0} :0±0.00a<br>10^{-1} :0±0.00a<br>10^{-2} :0±0.00a<br>Nts :0±0.00a<br>R: >7.08
Concentration of P2 63 g/100 mL	10^{-0} :0±0.00a<br>10^{-1} :0±0.00a<br>10^{-2} :0±0.00a<br>Nts :0±0.00a<br>R: >7.10	10^{-0} :0±0.00a<br>10^{-1} :0±0.00a<br>10^{-2} :0±0.00a<br>Nts :0±0.00a<br>R: >7.08
Concentration of P2 70 g/100 mL	10^{-0} :0±0.00a<br>10^{-1} :0±0.00a<br>10^{-2} :0±0.00a<br>Nts :0±0.00a<br>R: >7.10	10^{-0} :0±0.00a<br>10^{-1} :0±0.00a<br>10^{-2} :0±0.00a<br>Nts :0±0.00a<br>R: >7.08
Ethanol (E)		
Concentration of E 80 g/100 mL	10^{-0} :192±0.01a<br>10^{-1} :95±0.01b<br>10^{-2} :8.5±0.09ab<br>Nts :>100<br>R: >3.93	10^{-0} :192±0.03a<br>10^{-1} :80±0.08a<br>10^{-2} :6±0.06ab<br>Nts :>100<br>R: >3.93

Concentration of E 90 g/100 mL	10^{-0} :5.5±0.01a<br>10^{-1} :0±0.00a<br>10^{-2} :0±0.00a<br>Nts :<0.1<br>R: >7.11	10^{-0} :1.5±0.01a<br>10^{-1} :0±0.00a<br>10^{-2} :0±0.00a<br>Nts :0±0.00a<br>R: >7.11
Concentration of E 100 g/100 mL	10^{-0} :0±0.00a<br>10^{-1} :0±0.00a<br>10^{-2} :0±0.00a<br>Nts :0±0.00a<br>R: >7.11	10^{-0} :0±0.00a<br>10^{-1} :0±0.00a<br>10^{-2} :0±0.00a<br>Nts :0±0.00a<br>R: >7.11

Each value is the mean of three replicates with the standard deviation in three independent experiments. Any two means in the same column followed by the same letter are not significantly ($P > 0.01$) different by Tukey's multiple range tests,

* medium used: Trypticasein Soy LAB-Agar (TSA), neutralizer used: solution of Polysorbate 80 (3.0 g/100 mL), sodium thiosulphate (0.3 g/100 mL) and soy lecithin (0.3 g/100 mL), incubation conditions: 24h at 37 ± 1 °C, loading substance: bovine serum albumin (0.3 g/100 mL), the diluent used during the test: sterile hard water 30 mg/100g $CaCO_3$, test method and its validation: neutralization method for solutions, test temperature: 20 ± 1 °C, method of microbial counting: deepwell plate inoculation, stability of the preparation/diluent mixture: no precipitate formed during the test,

Nts - the number of units remaining after the test is performed,

R - reduction in the number of microorganisms during the test,

[a, b] - different letters: values differ significantly between the analyzed preparations.
